# The economics of TAVI: A systematic review

**DOI:** 10.1016/j.ijcha.2023.101173

**Published:** 2023-01-25

**Authors:** Panagiotis Petrou

**Affiliations:** a University of Nicosia, School of Sciences and Engineering, Pharmacy School, Pharmacoepidemiology-Pharmacovigilance, Nicosia, Cyprus; b University of Nicosia, Department of Life and Health Sciences, School of Sciences and Engineering, Pharmacoepidemiology-Pharmacovigilance, Nicosia, Cyprus

**Keywords:** Systematic review, Economic evaluation, Cost-effectiveness, Heart valve implantations, Transcatheter valve implantation, Surgical aortic valve replacement

## Abstract

**Objective:**

The scope of this systematic review is to update the existing body of evidence regarding the cost-effectiveness of transcatheter aortic valve implantation, stratified across all risk categories, and to assess their methodological quality.

**Methods:**

A systematic review was performed including published cost-effectiveness analyses of heart valve implantations. The quality was assessed with the Quality of Health Economics Tool.

**Results:**

We identified 33 economic evaluations of transcatheter aortic heart valve implantations. Results were not consistent, ranging from dominant to dominating. Moreover, the models were sensitive to an array of variables. The methodological quality of the studies was good.

**Conclusion:**

This systematic review led to inconclusive and inconsistent results pertinent to the economic profile of TAVI technology. It also highlighted areas which merit further research regarding the pillars of cost-effectiveness analysis such as modeling, the extrapolation of available data and the uncertainty of the evidence. A thorough assessment of the patient should proceed any decision-making.

## 1 Introduction

Aortic stenosis (AS) is the most common type of valvular heart disease in developed nations and its prevalence increases, owing to an ageing population. The surgical aortic valve replacement (SAVR) has been established as the cornerstone in symptomatic patients, since medical treatment alone perpetuates to a 12-month mortality rate in excess of 30 % [1], [2].

Despite the evolution in SAVR technology, a substantial percentage of patients are not eligible for surgery, since such a major operation would probably expose certain patients to an excessive risk–primarily, the ones with concomitant comorbidities, inability to undertake cardiopulmonary bypass due to aortic calcification and other health conditions. This percentage may surpass 30 % of all AS patients.

The introduction of transcatheter aortic valve implantation (TAVI), using either a percutaneous or a transapical approach, provided another viable therapeutic option for patients, whose health condition ruled out a major procedure. Since its advent in 2002 by Cribier et al [3], in an inoperable patient using a balloon-expandable valve, TAVI has profoundly reformed the aortic replacement landscape and has primarily emerged as the only option for inoperable patients. Currently, as more data are gleaned, the focus has expanded, apart from the inoperable and high-risk patients, to the intermediate and low-risk patients as well.

Currently, 5 valves are commercially available, however virtually all economic evaluations were performed with SAPIEN® Edwards and Evolut®Valve. SAPIEN® valve (Edwards Life Sciences, Irvine, CA, USA) is a balloon expandable valve, which consists of bovine pericardial leaflets supported within a tubular, slotted, stainless steel, balloon-expandable stent that is placed in the subcoronary position. Currently, the 5th version is commercially available. Evolut® System (Medtronic Inc., Minneapolis, MN, USA) is a self-expandable valve which consists of three porcine pericardial leaflets mounted in a self-expanding nitinol multi-staged frame. The use of nitinol with property of shape memory enables the use of a catheter delivery system and therefore a balloon is not essential. Apart from this distinct feature, they differ in the incidence of permanent pacemaker implantation, which favors SAPIEN®, and the delivery sheath as well. Evolut® uses an 18 French sheath, while SAPIEN® a 22 or 24, which explains the higher rate of vascular complication documented with the SAPIEN® valve [4]. SAPIEN® is available at 23, 26, and 29 mm and it can be implanted both through a transfemoral (TF) and a transapical (TA) route. The Evolut® is available in 23, 29, and 31 mm and it can be inserted only for the retrograde TF, subclavian approach, or direct aortic access.

The delivery mode is an important aspect of TAVI. The retrograde delivery is done via the femoral artery, while access through the subclavian artery and ascending aorta can be also considered. Unfavourable iliofemoral anatomy or extensive vascular disease is an indicator for a subclavian approach.

## 2 Risk evaluation in TAVI patients

The risk stratification of patients presenting with AS comprises a critical aspect regarding the selection of the suitable patient for TAVI. ESC in the most recent recommendations, considers TAVI in older patients (≥75 years), or in those who are high-risk (STS-PROM/EuroSCORE II > 8 %) or unsuitable for surgery. Non-transfemoral TAVI may be considered in patients who are inoperable for SAVR and unsuitable for transfemoral TAVI [5].

## 3 Costs

The most reckoned blueprint of the TAVI technology is their costs, which greatly -and negatively- deviates from the commensurate cost of surgical valves. The steadily increasing prevalence of AS, in the context of an ageing population, will stretch out the fiscal capacity of payers worldwide. These concerns are further exacerbated by the penetration of TAVI in other patient risk groups, apart from their established use in inoperable and high—risk patient cohorts. Data were only recently published for intermediate and low-risk patients and the body of evidence is currently being amassed. The concept of cost-effectiveness analysis has been the hallmark in decision-making and contribute in the allocation of health care resources, safeguarding equity and social cohesion in health care provision.

To this direction the scope of this paper is to critically assess and update the performed economic evaluations of TAVI across all risk cohorts, in order to provide guidance on the efficiency of this treatment modality. Economic evaluations can bring about significant insights and elucidate the full potential of each product, capitalizing on all variables.

## 4 Methodology

A systematic review was performed through March 2022 across PubMed, Medline, the Cochrane database, Embase, TCTMD, ClinicalTrials.gov, Clinical Trial Results, CardioSource, abstracts and presentations from major cardiovascular meetings. The Patient, Intervention, Comparator and Outcomes (PICO) criteria were set as following:

• Patients: Severe, Symptomatic aortic patients, which were classified as inoperable, high risk, medium or low surgical risk.
• Interventions: Implantation of transcatheter aortic valve
• Comparator: Medical management, surgical replacement or any other cardiac intervention
• Outcomes: Incremental cost-effectiveness ratio (ICER)/ life-year gained (LYG) and Incremental cost-utility ratio (ICUR) /quality adjusted life years (QALY)

We also searched for further articles cited by selected papers (snowball). The published proceedings and abstracts from important conferences and relevant proceedings, such as the American Heart Association, American College of Cardiology, Transcatheter Cardiovascular Therapeutics, Society of Cardiovascular Angiography and Intervention, European Society of Cardiology, and Euro-PCR, were also searched. In case of discrepancies, the authors were contacted. No restriction on publication dates were applied. We used the following combined search terms:

(“heart valve diseases”[MeSH Terms] OR (“heart”[All Fields] AND “valve”[All Fields] AND “diseases”[All Fields]) OR “heart valve diseases”[All Fields] OR (“valvular”[All Fields] AND “heart”[All Fields] AND “disease”[All Fields]) OR “valvular heart disease”[All Fields] OR (“aortic valve insufficiency”[MeSH Terms] OR (“aortic”[All Fields] AND “valve”[All Fields] AND “insufficiency”[All Fields]) OR “aortic valve insufficiency”[All Fields] OR (“aortic”[All Fields] AND “regurgitation”[All Fields]) OR “aortic regurgitation”[All Fields]) OR (“aortic valve stenosis”[MeSH Terms] OR (“aortic”[All Fields] AND “valve”[All Fields] AND “stenosis”[All Fields]) OR “aortic valve stenosis”[All Fields] OR (“aortic”[All Fields] AND “stenosis”[All Fields]) OR “aortic stenosis”[All Fields])) AND (“economics”[MeSH Subheading] OR “economics”[All Fields] OR “cost”[All Fields] OR “costs and cost analysis”[MeSH Terms] OR (“costs”[All Fields] AND “cost”[All Fields] AND “analysis”[All Fields]) OR “costs and cost analysis”[All Fields] OR (“cost benefit analysis”[MeSH Terms] OR (“cost benefit”[All Fields] AND “analysis”[All Fields]) OR “cost benefit analysis”[All Fields] OR (“economic”[All Fields] AND “evaluation”[All Fields]) OR “economic evaluation”[All Fields]) OR (“cost benefit analysis”[MeSH Terms] OR (“cost benefit”[All Fields] AND “analysis”[All Fields]) OR “cost benefit analysis”[All Fields] OR (“cost”[All Fields] AND “effectiveness”[All Fields]) OR “cost effectiveness”[All Fields]) OR (“cost benefit analysis”[MeSH Terms] OR (“cost benefit”[All Fields] AND “analysis”[All Fields]) OR “cost benefit analysis”[All Fields] OR (“cost”[All Fields] AND “benefit”[All Fields]) OR “cost benefit”[All Fields]) OR “cost utility”[All Fields]).

**Fig. 1:**
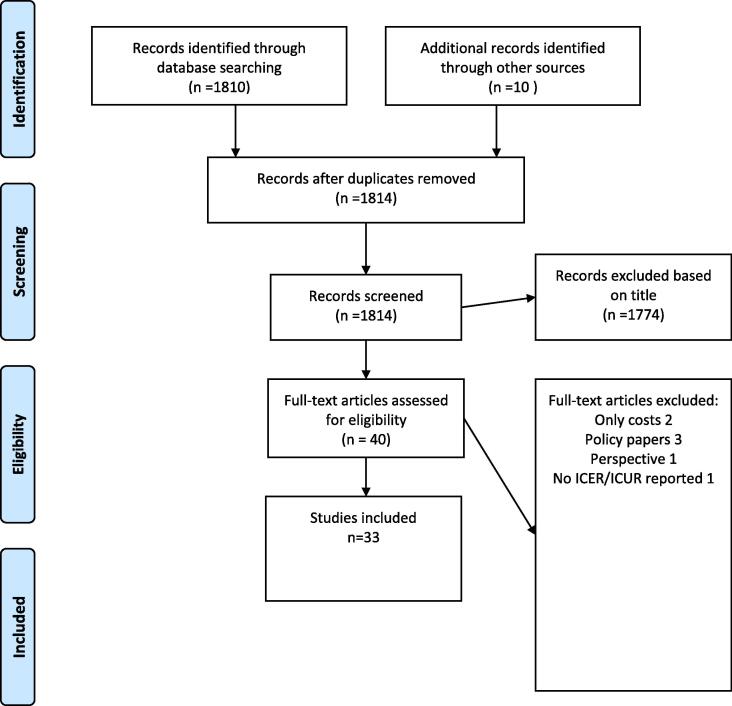
Flow diagram of literature review.

The searched yielded 1814 results. We eliminated duplicates and after an initial screening, 40 studies remained. The final set consists of 33 studies. (Fig. 1).

The eligibility criteria were set as following:

1) Studies should include a complete economic evaluation of valve replacement;
2) Studies should report the ICUR and/or ICER as either cost per quality-adjusted life year (QALY) gained or as cost per life-year gained (LYG) or both.

We adhered to the Preferred Reporting Items for Systematic Reviews and meta-Analysis approach (PRISMA) [6].

## 5 Data extraction and critical appraisal

Each study was independently reviewed by two reviewers. In case of discrepancies, the lead reviewer was consulted and a consensus was pursued. For each study we extracted the following information: Authors, Year, Perspective (societal, payer, provider) and Country, comparators, time horizon, model, outcomes and their source, discounting, funding, effectiveness, costs, incremental costs and WTP threshold. The lead reviewer also verified the correctness of the final data set. The quality was assessed through the QHES questionnaire [7]. QHES is a validated tool and it is made up of 16 weighted points. The QHES instills value by elaborating a qualitative analysis of the results of assessment of individual items. The causal hypothesis is that higher-quality studies will perpetuate to a better decision-making framework, by tackling bias and misuse [8]. After applying the predetermined selection criteria, 24 studies remained for the final analysis. A narrative review was adopted.

## 6 Results

### 6.1 Inoperable patients

Kodera et al compared TA or TF TAVI with SAPIEN valve implantation and medical management (MM), based on the results of the PARTNER cohort B [9], [10]. The time horizon was set at a 10-year time frame and by that period the anticipated costs of TAVI were estimated at ¥8,014,886 JPY, while medical therapy was associated to ¥1,639,824 JPY, leading to an incremental cost of ¥6,375,062 JPY. This was largely offset by the health gain, which was estimated at 3.02 and 1.27 QALYs for the TAVI and medical therapy, respectively. The ICUR of TAVI compared with medical therapy was ¥3,918,808 yen per QALY gained, which is below the Japanese WTP threshold.

The study of Kodera bears a close resemblance with a subsequent study by Inoue et al, who performed an analysis of inoperable patients from a Japanese payer perspective. A model consisting of a decision analytic model for two years and then a Markov model was used to elucidate the ICER in this patient cohort. For the cohort of inoperable patients, the PARTNER 1 and 2 trials were utilized [10], [11], [12]. TAVI was associated with ¥7,654,277JPY while SAVR with ¥816,682JPY. TAVI created 3.26 QALY while SAVR 1.28 QALY per patient, leading to an ICER of ¥3,460,810, which classify the treatment as a cost-effective therapeutic modality in Japan.

Hancock et al published an economic evaluation of TAVI vs MM in inoperable patients, from a Canadian payer perspective. They used a deterministic decision analytic model over a 3-year time horizon. The efficacy data and utility were mined from the PARTNER trial [10], [13]. Costs were set from a Canadian payer perspective. TAVI yielded 1.325 QALYs per patient, while MM resulted in 0.837 QALYs per patient, which leads to an incremental increase of 0.49 for TAVI. The gain came at an incremental cost of CAD$15,687 (TAVI CAD$58,357 MM CAD$42,670). These spiral to an ICUR of CAD$32,170 per QALY. Model was sensitive to the time horizon of the study [13].

Watt et al elaborated on a decision analytical model with a 10-year horizon, which used efficacy and utility data from the PARTNER cohort B [10]. The costs were set from the NHS perspective. Over the 10-year horizon, TAVI incurred an incremental cost of £25,200 vs MM, which was partially offset by an additional 1.56 QALYs gained in TAVI patients. Therefore, the estimated ICUR was £16,200 per QALY gained, which is considered cost-effective in UK perspective. Model was sensitive to variations of the short-term treatment effect and the initial operation cost [15].

Another study hailing from UK assessed the cost-effectiveness of TAVI in inoperable patients from the perspective of the English payer. Murphy et al employed a decision tree Markov Model with a lifetime horizon [16]. The efficacy data were mined from the PARTNER B cohort. The utilities were based on the estimations presented by Maliwa et al [17] and costs were set on the perspective of the British NHS. Authors concluded that TAVI led to more effectiveness compared to MM (1.63 versus 1.19 QALYS and 2.54 versus 2.24 life-years gained). This came at a significant incremental cost (£28,061 versus £12,176) and the estimated ICUR was £35,956 per QALY. The model was sensitive to short- and long-term transition probabilities for the TAVI arm. The reported ICUR slightly exceeded the endorsed cost-effectiveness threshold in the UK (£20,000–£30,000 per QALY).

Simons estimated the cost-effectiveness of TAVI vs medical management from a US payer perspective. TAVI conferred 2.9 years life expectancy while it also delivered 0.73 more QALY. TAVI incurred USD$85,600 more discounted cost. The incremental cost-effectiveness ratio (ICUR) of TAVI compared with medical management was USD$99,900 per life-year gained and USD$116,500 per QALY gained. Model was sensitive to other, non-aortic stenosis related medical costs, which are related to comorbidity issues of patients [18].

Brecker performed an economic evaluation of TAVI vs MM in inoperable patients from the perspective of the UK NHS [19]. They created two Markov model, one for short-term (30 days) and one for long-term assessment. The efficacy data were mined from the ADVANCE trial [20]. The ICUR of TAVI versus medical treatment was £13,943 per QALY. A subset analysis based on the ADVANCE study’s high-risk cohort (Logistic EuroSCORE > 20 %) led to higher ICUR was £17, 718 per QALY. The model was most sensitive to the cost of HF hospitalizations in the MM cohort.

Pinar et al evaluated the cost-effectiveness of SAPIEN 3 in an inoperable Spanish population, through the use of a Markov Model with 9 exclusive states, at a 15-year life horizon. The efficacy data were mined from the PARTNER trial and the utilities from the PARTNER 2 trial. Costs were set from the perspective of the Spanish payer. Authors concluded that the TAVI was associated with superior results (LYG 3.17 vs 1.60 QALYs 2.09 vs 0.78) and higher costs as well. These led to an ICER of €3,454/LYG and ICUR of €4,169/QALY. Outcomes were sensitive to the time horizon [21].

Doble et al studied the cost-effectiveness of TAVI from a Canadian third-party payer’s perspective over a 20-year horizon. They efficacy and utility data were extracted from the PARTNER B cohorts. Doble concluded that the forecasted costs of TF-TAVI, over the 20-year horizon exceeded the corresponding ones of MM (CAD$88,991 and CAD$57,963 respectively). TAVI was also superior, both in terms of LYG and QALY (0.85 LYG and 0.6 QALY respectively. The ICER was estimated at CAD$36,458/LYG and the ICUR at CAD$51,324/QALY, respectively. The model was most sensitive to the costs of the interventions and the 1-year mortality rates for both treatments. The rates of paravalvular leaks and 30-day mortality for the TF-TAVI treatment were also major determinants of the outcome of the economic evaluation [22].

Reynolds analyzed the economic profile of SAPIEN along the clinical trial PARTNER B. Based on the one-year collected data, it was projected that under a lifetime horizon, TAVI would deliver an incremental gain of 1.6 LYG and 1.3 QALY, which perpetuate to an ICER of USD$50,200/ LYG and an ICUR of USD$61,889/QALY [23].

Lorenzoni et al assessed the cost-effectiveness of SAPIEN, from the perspective of the Italian National Health System (INHS) [24]. Regarding the inoperable patient cohort, a Markov model was created which was fed with the relevant clinical events were derived from Herrmann et al and Leon et al [10], [11], [12], [13], [14], [15], [16], [17], [18], [19], [20], [21], [22], [23], [24], [25]. Costs were set from the perspective of the Italian Health care system and were discounted 3 % yearly, along with outcomes. TAVI delivered 1,83 QALY vs 0,65 of ΜΜ, at an incremental cost of 11,919 EUR. TAVI was also associated with an incremental gain of 1,57 LYG. These led to an ICUR of €10,133/QALY and an ICER of €7,577/LYG. Model was sensitive to mortality, re-operation risk for SAVR, risk and costs of hospitalizations for heart failure (HF) and valvuloplasty.

**Fig. 2:**
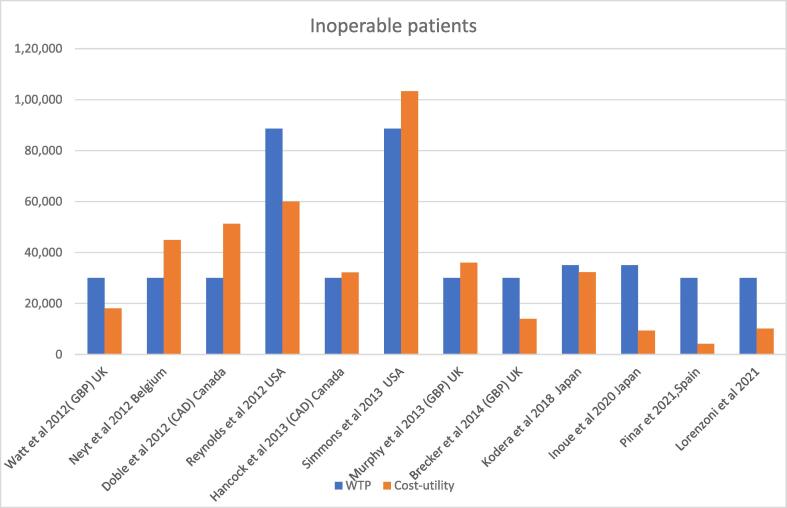
Cost-effectiveness results in inoperable patients.

In 2012, Neyt reported on the economic evaluation of TAVI in operable patients, from the perspective of the Belgian Health Care System. Based on the PARTNER trial, they deduced that in inoperable patients, and assuming a lifetime scenario, TAVI conferred more health gains, albeit a a higher cost. The Incremental gain in LYG was 0.74 while the corresponding in QALY was 0.88 QALY, which spiraled to an ICER of €42.600 per LYG and an ICUR of €44.900 per QALY [26]. (See Table 1 and Fig. 2).

**Table 1:** Economic evaluations in inoperable patients.

**Author publication year)**	**Country/perspective/Funding**	**Model**	**Agent**	**Time Horizon**	**Treatment Incremental costs**	**Currency**	**LYG gained**	**QALY’s gained**	**Cost-effectiveness (CE) LYG**	**WTP**	**Cost-utility (CU) QALY**	**Other comments**
Lorenzoni et al, 2021 [24]	Italy/Italian Health System Perspective/ Industry funded	A Markov model with a 1-month cycle length	SAPIEN 3 vs MM	15-year horizon.	€11,919	EUR	Incremental gain: 1.57 LYG TAVI:3.17 LYG MM:1.60 LYG	Incremental gain: 1.18 QALY TAVI:1.83 QALY MM:0.65 QALY	€7,577 per LYG	€30,000	€10,133 per QALY gained	Model was sensitive mortality, re-operation risk for SAVR, risk and costs of hospitalizations for HF and valvuloplasty.
Pinar et al, 2021 [21]	Spain/National Health System Perspective/Industry sponsored	Markov model (monthly cycles) with 8 states	SAPIEN 3 vs MM	15-year horizon	Incremental costs: €5,434 TAVI: €40, 250 MM:€34, 816	EUR	Incremental gain:0.57 LYG TAVI 3.17 LYG MM: 1.60 LYG	Incremental gain:1.31 QALY TAVI:2.09 QALY MM:0.78 QALY	€3,454 per LYG	€30,000	€4,169 per QALY gained	Model was sensitive to the time horizon
Inoue et al, 2020 [11]	Japan/ Japanese Public Healthcare Payer Perspective/ Industry Funded	Markov Model	TF TAVI SAPIEN XT vs SoC	Lifetime horizon	TAVI ¥7,654,277 SoC ¥816,682	JPY	N/R	Incremental gain:1.9758 QALY TAVI: 3. 2607 QALY SoC:1.2849 QALY	N/R	¥5,000,000	ICUR TF-TAVI versus SOC ¥3.5 million perQALY.gained	Results were sensitive to the utility values of no event survival
Kodera et al,2018 [9]	Japan/Japanese Public Healthcare Payer Perspective/ No Funding reported	Markov model with Monte Carlo simulations	TF or TA TAVI vs MM	10-year horizon	Incremental costs: ¥6,375,062 TF TA TAVI:¥ 8,014,886 MM:¥ 1,639,824	JPY	N/R	Incremental gain:1.75 TF TA TAVI: 3.02 QALY MM: 1.27 QALY	N/R	¥5,000,000	¥3,918,808 per QALY gained	Results were sensitive to the long-term mortality of TAVI and the acquisition cost
Brecker et al, 2014 [19]	UK/NHS Perspective/ Industry Funded	Decision-analytic model	TAVI vs MM	5-year horizon	Incremental costs:£21,038 TAVI: £34,192 MM: £13,154	GBP	N/R	Incremental gain:1.51 QALY TAVI:2.29 QALY MM:0.78 QALY	N/R	£30,000	£13,943 per QALY gained	Model was sensitive to the HF hospitalisation costs of the MM cohort
Hancock et al, 2013 [14]	Canada/ Canadian Public Healthcare system Perspective / Industry Funded	Deterministic decision analytic model	TAVI vs MM	3-year horizon	Incremental cost: CAD$15,687 TAVI:CAD$58,357 MM CAD$42,670	CAD	N/R	Incremental gain: 0.49 QALY TAVI: 1.325 QALY MM: 0.837 QALY	N/R	CAD$20,000–$100,000	CAD$32,170 per QALY gained	Model was sensitive to the time horizon
Murphy et al 2013 [16]	UK NHS Perspective / Industry funded	The model employed two parts, a short-term decision tree spanning over the first month and a longer-term Markov model which covered the period from 30 days to death.	TAVI vs MM	1 year	Incremental cost:£15,885 TAVI:£28,,061 MM:£12,176	GBP	Incremental gain:0.3 LYG TAVI:2.54 LYG MM:2.24 LYG	Incremental gain:0.44 QALY TAVI:1.63 QALY MM:1.19 QALY	N/R	£30,000	£35,956 perQALY gained	The model was sensitive to procedure related events
Simons et al 2013 [18]	USA/Societal Perspective / Funded by Public sector	Markov model	TF TAVI vs MM	Lifetime	Incremental cost: $85,600 TAVI: $169,100 MM: $86,300	USD	Incremental gain:0.86 LYG TAVI: 2.93 LYG MM:2.08LYG	Incremental gain: 0.73 QALY TAVI: 1.93 QALY MM:1.19 QALY	$99,900 per LYG	$100,000	$116,500 per QALY gained	The results were sensitive to the level of annual healthcare costs unrelated to aortic stenosis
Neyt et al 2012 [26]	Belgium/Belgian Health System Perspective /No funding was received	Markov model	TF TAVI SAPIEN vs SoC	Lifetime	Incremental costs: €36,887 TAVI:€40,057 SoC: €3,170	EUR	Incremental gain:0.74 LYG	Incremental gain:0.88 QALY	€42,600 per LYG		€44,900 per QALY gained	
Reynolds et al 2012 [23]	USA/US modified societal healthcare system Perspective//Industry funded	Bootstrap resampling	SAPIEN TAVI vs SoC	Lifetime horizon	Incremental costs: $52,455 TAVI: $106,076 MM: $53,621	USD	Incremental gain 1.3 LYG TAVI: 2.5 LYG MM: 1.2 LYG	Incremental gain:1.59 QALY TAVI:2.78 QALY MM:1.2 QALY	$50,212 per LYG	≈$70,000	$61,889 per QALY gained	
Watt et al 2012 [15]	UK/ NHS Perspective/ Industry Funded	two interlinked Markov models	TF TAVI SAPIEN vs MM	10-year horizon	Incremental costs:£25,200 TAVI: £30,200 MM: £5,000	GBP	N/R	Incremental gain:1.56 QALY TAVI;2.36 QALY MM:0.8 QALY	N/R	£20, 000–£30, 000	£16,200 per QALY gained	The model was very sensitive to changes to the short-term treatment effect and the cost of the initial operation
Doble et al. 2012 [22]	Canada/Third-party Canadian health-care payer /HTA Funded	A combined decision tree and Markov model	TF TAVI SAPIEN vs MM	20-year horizon	Incremental costs: CAD$31,198 TAVI: CAD $88.891 MM:CAD$57,693	CAD	Incremental gain:0.85 LYG	Incremental gain:0.6 QALY	CAD$36,458 per LYG	CAD$50,000	CAD$51,324 per QALY gained	The model was most sensitive to the procedural costs and 1-year mortality rates for both treatments. The rates of paravalvular leaks and 30-day mortality for the TF-TAVI treatment were also sensitive to change.

Abbreviations: MM, medical management; SoC, Standard of care; TF, transfemoral; TA, transapical; LYG, life-years gained; QALY, quality -adjusted life years.

### 6.2 High risk patients

Inoue et al published their findings pertinent to the economics of TAVI from a Japanese payer perspective [11]. They employed a decision tree and Markov model, based on the SOURCE XT registry [27] and the SAVR arm of the CoreValve US pivotal trial [28], respectively. Inoue concluded that in Japan TAVI costs more but also delivers more utility than SAVR. These perpetuates to an ICUR of ¥1,300,000 JPY/QALY. In contrast to Kodera, whose sensitivity analysis ranked first the long-term mortality rate of TAVI, Inoue posited that the results were sensitive to long-term survival following SAVR.

Doble et al explored the cost-effectiveness of TAVI from a Canadian third-party payer’s perspective, over a 20-year horizon [22]. The efficacy and utility data were extracted from the PARTNER B cohorts. Doble summarized that the forecasted costs of TAVI, over the 20-year horizon exceeded the corresponding ones of SAVR ($85,755 CAD and $74,602 CAD). TAVI yielded an incremental LYG (0.0128) compared to SAVR. This spirals to an ICER of $870,143/LYG. Nevertheless, SAVR dominated TAVI, meaning it was less costly and more effective, in terms of QALY since it conferred 0.102 less QALYs.

Fairbairn evaluated the economic profile of TAVI vs SAVR from a UK perspective, through a Markov model with a 10-year horizon [29]. The efficacy data were transferred from the PARTNER A study while the utility data were generated from a UK study population previously described. At the 10-year threshold, a modest QALY gain was observed, in favor of TAVI (2.81 and 2.75, respectively). The cost for SAVR were marginally higher compared to the corresponding ones for TAVI (£19,368 for TAVI and £20,380 for SAVR). Consequently, TAVI dominated SAVR.

Freeman investigated the cost-effectiveness of TAVI using real-world data from a patients’ cohort through a decision analytic model, compared to standard care. Nevertheless, the baseline characteristics between the two groups diverged, which could have confounded the results. The efficacy data were derived from a sample of 155 local patients. The utility values were quantified based on the results of the PARTNER B study. TAVI produced more incremental QALY (1.29) and more LYG (1.73) compared to standard care. These gains came at an incremental cost of £ 13,655 which delivered an ICUR of £10,533/QALY [30].

Gada studied the economics of transapical aortic valve implantation in high-risk patients vs aortic valve replacement and medical management from the perspective of a US payer. The created a Markov Model with a lifetime horizon [31]. They utilized efficacy data from a US registry [32] while utility values were calculated based on the Medical Expenditure Panel Survey[33]. Gada et al concluded that TAVI rendered an ICUR of $44,384 vs SAVR and $42,637 vs medical management. SAVR dominated TAVI. Their model was sensitive to variations in the probabilities of *peri*-operative, annual mortality after each intervention, and the annual probability of stroke following AVR.

The same team performed an economic evaluation of TF valve implantation (SAPIEN). They used the same model assuming the same variables. Authors concluded that TAVI yielded an ICUR of $52,773/QALY compared to SAVR. The second scenario conferred an ICUR of $32,000/QALY. The use of the PARTNERT A data exerted a beneficial effect on the ICUR, containing it to $32,000/QALY. Their third scenario, which utilized PARTNER cohort A data (transition probabilities, mortality rates, and costs) the clinical difference between TAVI and SAVR was marginal, which explains the steep ICUR of $252,400/QALY. Model was sensitive to perioperative and annual mortality after SAVR and after TAVI, and the probability of annual stroke after TAVI [34].

Povero et al determined the economic aspects of TAVI vs Perceval® bioprosthesis valve in intermediate to high-risk patients [35]. Model. The lifetime simulation study was performed from the perspective of six countries, namely US, Germany, UK, France, Italy, and Australia. Authors proceeded with a patient-level discrete event simulation (DES) structure for the in-patient care, while the lifetime scenario was evaluated through the use of a cohort Markov model with monthly cycles. The efficacy data were pooled from several studies while the in-hospital care data were mined from the SOURCE 3 registry [36]. A 3.5 % annual discount rate were applied to both costs and outcomes after hospital discharge. The studies of Ussia [37] fitted the calculation of hospitalization free survival. The mild-to-severe paravalvular leak (PVL) which comprises an independent predictor of overall survival (OS) after TAVIs or SAVR was calculated based on the Kodali et al [38]. Authors concluded that sutureless valve replacement dominates TAVI, a consistent finding across all studied countries. Model was sensitive to PVL, time horizon and the efficacy discount rate.

Neyt et al proceeded to assess the cost-effectiveness of TAVI from a Belgium payer-perspective. A Markov model was based on the efficacy data of the PARTNER study which assumed transfemoral or transapical implantation of TAVI. A one-year horizon was used. During the first year, TAVI patients gained 0.03 QALYs compared to SAVR patients. This marginal gain, coupled with the incremental costs (€20,397) spiraled to an ICUR which surpassed €750,000/QALY and rules-out any probability of the procedure being cost-effective. Model was sensitive to mortality and utility rates [26].

The cost-effectiveness of SAPIEN, from the perspective of the Italian National Health System (INHS) was the core of Lorenzoni’s study. Regarding the high-risk patient cohort, a Markov model was created which was fed with survival data from the studies of Herrmann et al. [25] and Mack et al. [39] for TAVI and SAVR, respectively, were used for high-risk patients. Costs were set from the perspective of the Italian Health care system and were discounted 3 % yearly, along with outcomes. TAVI delivered 2.83 vs 2.48 QALY compared to SAVR, at an incremental cost of €3,831. TAVI was also associated with an incremental gain of 0.4 LYG. These led to an ICUR of £11,209/QALY and an ICER Of £9,474/LYG. Model was sensitive to mortality, to major incidence of stroke and repeated hospitalizations for AS.

Pinar et al studied the economics of SAPIEN 3 TAVI from the Spanish Payer perspective [21]. A Markov mathematical model with 9 exclusive states with monthly cycles was used based on the PARTNER 1 trial [40]. Model had a 15-year time horizon and the utilities were extracted from the PARTNER 2 trial. Costs were set from the perspective of the Spanish health care system and they were discounted 3 % annually, along with benefits. TAVI yielded €49,346, €2,155 more than the commensurate ones of s AVR. The total LYG per TAVI patient was 4.49, as compared to 4.08 of SAVR patients, while the QALY gain was superior in TAVI patients (3.13 vs 2.74 QALY). The ICER perpetuates to €5,329/LYG and ICUR was €5,471/QALY.

Reynolds [40] performed an economic evaluation of the SAPIEN 3 valve from a US payer perspective. Their approached used the PARTNER trial data, under one year horizon. Due to distinct differences between transfermoral and transapical implantation, the two approaches were presented individually. In the entire sample, TAVI was superior to SAVR, both in terms of QALY (0.633 vs 606) and LYG gains (0.858 vs 0.817). This superiority was echoed in the transfemoral cohort (LYG 0.878 vs 0.813 and QALYs (0.659 vs 0.591) but not in the transapical (TA) cohort. In the latter, SAVR dominated TAVI since it was superior (LYG 0.811 vs 0.826 and QALYs 0.570 vs 0.641). Overall, the ICUR of TAVI was $76,877/QALY. Of interest is the fact that TAVI dominated SAVR in the TF cohort, while on the contrary, it was dominated by SAVR pertinent to the TA. cohort A second study hailing from the same author assessed the economic profile of self-expanding valve in a US population. The same trial-based approach, under one year horizon was implemented[41]. TAVI yielded an incremental lifetime gain of 0.32 QALY. Lifetime incremental cost-effectiveness ratios were $55,090 per QALY gained and $43,114 per LY gained. Model was sensitive to the cost of TAVI.

Geisler [42] in 2007 published a study referring to the economic profile of the Evolut valve vs SAVR in a Dutch, high-risk, population. They created a model based on the CoreValve High Risk Trial[43], which provided both efficacy and utility values as well. Costing was set as per Dutch payer perspective. TAVI was related with an 0.65 incremental LYG (5.62 for TAVI vs 4.97 for SAVR) and 0.41 QALYs (3.69 for TAVI vs 3.27 for SAVR). The incremental cost was also higher for TAVI €9,048 (€51,068 vs €42,020). This led to an ICUR of €21,946 per QALY gained. The model was sensitive to procedure time and hospitalization length of stay.

Tarride et al reported the findings of the economic profile of TAVI from a Canadian health care perspective [44]. They created a Markov Model with 9 mutually exclusive health states, comparing the SAPIEN 3 with SAVR, over a 15-year horizon. The model was loaded with the PARTNER 1A results and the utility studies from the PARTNER trial. The TAVI cohort reported more total costs (CAD$70,497 vs CAD$65,507) compared to the SAVR, while it delivered more QALYs (3.57 vS 3.15). This led to an ICUR of CAD$17,237/QALY. The results were sensitive to the time-horizon of the study.

**Fig. 3:**
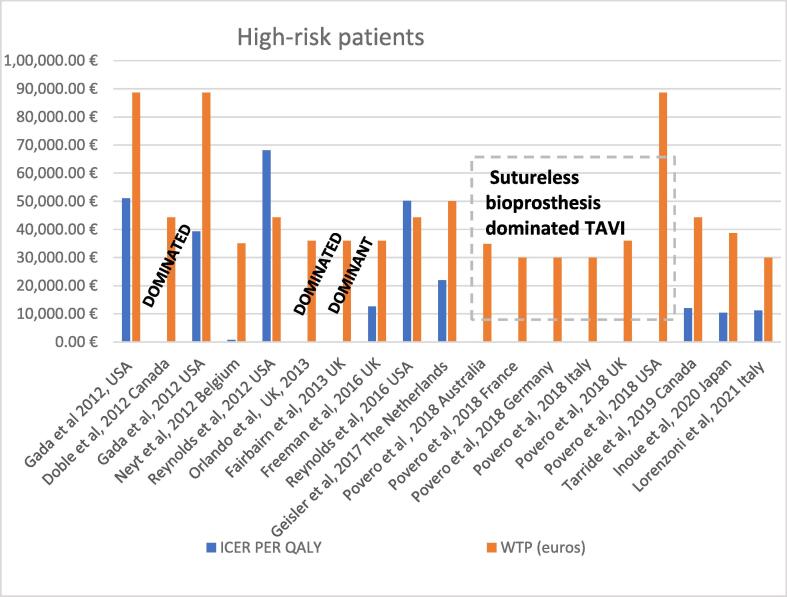
Cost-effectiveness results in high-risk patients.

Orlando performed an economic evaluation of TAVI from the perspective of the NHS in UK. They created a decision tree based on the PARTNER trial and they concluded that TAVI was dominated by SAVR [45]. (See Table 2 and Fig. 3).

**Table 2:** Economic evaluations in high-risk patients.

**Author publication year)**	**Country/Perspective /Funding**	**Study Model**	**Access**	Agents	**Time Horizon**	**Treatment Incremental costs**	**Currency**	**LYG gained**	**QALY’s gained**	**Cost-effectiveness (CE) LYG**	**WTP**	**Cost-utility (CU) QALY**	**Comments**
Lorenzoni et al 2021 [24]	Italy/Italian health system perspective/ Partially funded from industry	Markov Model with 8 different health states	NR	TAVI SAPIEN 3 vs SAVR	15-year time horizon	Incremental costs: €3,831 TAVI: €37,189 SAVR: €33,358	EUR	Incremental gain: 0.41 LYG TAVI: 4.49 LYG SAVR: 4.08 LYG	Incremental gain:0.34 QALY TAVI:2.83 QALY S AVR:2.49	€9,474 per LYG	€30,000	€11,209 per QALY gained	Model was sensitive to mortality, to major incidence of stroke and repeated hospitalizations for AS.
Inoue et al, 2020 [11]	Japan/Japanese Public healthcare Payer perspective/ Industry Funded	Decision tree model for the first two years and a Markov model	TA/TF	TAVI SAPIEN XT vs SAVR	Lifetime	Incremental costs:¥1,556,750 TAVI:¥ 7, 725,818 SAVR: ¥6,169, 068	JPY	N/R	Incremental gain: 1.19 QALY TAVI: 5.558 QALY SAVR: 4.3946 QALY	N/R	¥ 5,000,000 .	¥1,337,52_5_ per QALY gained	Model was sensitive to mortality of SAVR patients after 24 months
Tarride et al 2019 [44]	Canada/ Canadian third-party payer perspective/Industry Funded	Markov Model with 9 exclusive states	NR	TAVI SAPIEN 3 vs SAVR	15-year time horizon	Incremental costs: CAD$7,362 TAVI:CAD$84,348 SAVR: CAD$76,986	CAD	N/R	Incremental gain:0.42 QALY TAVI: 3.57 QALY SAVR: 3.15QALY	N/R	CAD$50.000	CAD$ 17,237 per QALY gained	Model was sensitive to the time horizon
Doble et al, 2012 [22]	Canada/ Third-party Canadian health-care payer/Funded	A combined decision tree and Markov model	TAVI (transfemoral or transapical) vs SAVR	TAVI SAPIEN vs SAVR	20 years	Incremental Costs:CAD$11,153 TAVI: CAD $85,755 SAVR:CAD $74,602	CAD	Incremental gain: 0.012 LYG	Incremental gain: 0.102 QALY	ICER CAD$870,143 per LYG	CAD$50,000	SAVR dominated TAVI	
Povero et al, 2018 [35]	Australia/ Third-party payer perspective/ Industry Funded	Patient-level discrete event simulation (DES) structure, wit a cohort Markov model	NR	TAVI vs Sutureless bioprosthesis Perceval ®	Lifetime	Incremental costs:–$10,016	AUD	Incremental gain:-1.25 LYG TAVI: 4.26 LYG SU AVR: 5.51 LYG	Incremental gain:-1.14QALY TAVI: 3.44 QALY SU AVR: 4.58 QALY	TAVI WAS DOMINATED	AUD $50,000	TAVI WAS DOMINATED	Model was sensitive to PVL, time horizon and efficacy discount rate.
Povero et al, 2018 [35]	France/ Third-party payer perspective/ Industry Funded	Patient-level discrete event simulation (DES) structure, wit a cohort Markov model	NR	TAVI vs Sutureless bioprosthesis Perceval®	Lifetime	Incremental costs:–€3,504	EUR	Incremental gain: −1.25 TAVI: 4.26 SU AVR: 5.51	Incremental gain:-1.14QALY TAVI: 3.44 QALY SU AVR: 4.58 QALY	TAVI WAS DOMINATED	€30,000	TAVI WAS DOMINATED	Model was sensitive to PVL, time horizon and efficacy discount rate.
Povero et al, 2018 [35]	Germany / Third-party payer perspective/Industry Funded	Patient-level discrete event simulation (DES) structure, wit a cohort Markov model		TAVI vs Sutureless bioprosthesis Perceval®	Lifertime	Incremental Costs:–€6,772	EUR	Incremental gain: −1.25 TAVI: 4.26 SU AVR: 5.51	Incremental gain:-1.14QALY TAVI: 3.44 QALY SU AVR: 4.58 QALY	TAVI WAS DOMINATED	€45,000	TAVI WAS DOMINATED	Model was sensitive to PVL, time horizon and efficacy discount rate.
Povero et al, 2018 [35]	Italy/ Third-party payer perspective /Industry Funded	Patient-level discrete event simulation (DES) structure, wit a cohort Markov model	NR	TAVI vs Sutureless bioprosthesis Perceval®	Lifetime	Incremental Costs:–€6,570	EUR	Incremental gain: −1.25 TAVI: 4.26 SU AVR: 5.51	Incremental gain:-1.14QALY TAVI: 3.44 QALY SU AVR: 4.58 QALY	TAVI WAS DOMINATED	€30,000	TAVI WAS DOMINATED	Model was sensitive to PVL, time horizon and efficacy discount rate.
Povero et al, 2018 [35]	UK/ Third-party payer perspective/ Industry Funded	Patient-level discrete event simulation (DES) structure, wit a cohort Markov model	NR	TAVI vs Sutureless bioprosthesis Perceval®	Lifetime	Incremental costs:–£7,991	GBP	Incremental gain: −1.25 LYG TAVI: 4.26 LYG SU AVR: 5.51 LYG	Incremental gain:-1.14QALY TAVI: 3.44 QALY SU AVR: 4.58 QALY	TAVI WAS DOMINATED	£30,000	TAVI WAS DOMINATED	Model was sensitive to PVL, time horizon and efficacy discount rate.
Povero et al, 2018 [35]	USA/ Third-party payer perspective/ Industry Funded	patient-level discrete event simulation (DES) structure, wit a cohort Markov model	NR	TAVI vs Sutureless bioprosthesis Perceval®	Lifetime	Incremental costs:–$20,930	USD	Incremental gain: −1.25 LYG TAVI: 4.26 LYG SU AVR: 5.51LYG	Incremental gain:-1.14 QALY TAVI: 3.44 QALY SU AVR: 4.58 QALY	TAVI WAS DOMINATED	US$100,000	TAVI WAS DOMINATED	Model was sensitive to PVL, time horizon and efficacy discount rate.
Geisler et al 2017 [42]	The Netherlands / Health care perspective / Industry Funded	Markov Model	NR	TAVI EVOLUT vs SAVR	Lifetime	Incremental costs:€9,048 TAVI €51,068 SAVR €42,020	EUR	N/R	Incremetnal gain: 0.42 QALY TAVI: 3.69 QALY SAVR :3.27 QALY	N/R	AS has a disease burden 0.43. For a disease burden between 0.41 and 0.70 the appropriate cost-effectiveness threshold is €50,000/QALY in the Netherlands	€21,946 per QALY gained	Model was sensitive to procedure time and hospital stay
Reynolds et al.2016[41]	USA/Societal perspective/ Industry Funded	Trial-based	TF/TA	TAVI vs SAVR	Lifetime	Incremental costs:$17,849 TAVI: US$207,478 SAVR: US$189, 629	USD	Incremental gain:0.41 LYG TAVI:5.469 LYG SAVR:5.055 LYG	Incremental gain:0.324 QALY TAVI:4.149 QALY SAVR:3.825 QALY	US$43,114/LYG	$50, 000	US$55090 per QALY gained	Model was sensitive to the cost of TAVI
Freeman et al 2016[30]	UK/UK NHS/ Funding not reported	Decision analytic model	N/R	TAVI vs MM	5-year time horizon	Incremental costs:£13,655TAVI: £44,751 MM: £31,096	GBP	Incremental gain:1.73 LYG	Incremental gain: 1.29 QALY		£20, 000–£30,000	£10,533 per QALY gained	
Fairbairn et al. 2013[29]	UK/ UK NHS perspective/ Funded by the British Heart Foundation	Decision tree and Markov model	TF/TA	TF/TA TAVI vs SAVR	10-year time horizon	Incremental costs--£1,350.38 TAVI: £52,593.02 SAVR: £53,943.40	GBP,	N/R	Incremental gain:0.06 QALY TAVI:2.81 QALY SAVR:2.75 QALY	N/R	£20,000–£30, 000	TAVI dominated SAVR	Model was sensitive to the cost of the tavi and the length of stay following the TAVI procedure.
Orlando et al 2013 [4 5]	UK/NHS perspective/ Funded by HTA programme	Decision tree	TF/TA	TAVI vs SAVR	25-year time horizon	Incremental Costs: £7,962 TAVI: £2,833 SAVR: £19,871	GBP,	N/R	Incremental gain:- 0.51 QALY TAVI: 2.85 QALY SAVR: 3.46 QALY	N/R	£20, 000–£30, 000	TAVI is dominated	
Gada et al. 2012[31]	USA/ Thid party payer perspective/ Funding not reported	Single Markov model	TA	TAVI SAPIEN vs MM and SAVR (		Incremental costs:$100 TAVI: US$56, 730 AVR: US$56, 630	USD	N/R	Incemental gain: −0.03 QALY TAVI:1.66 QALY SAVR:1.7 QALY	N/R	US$100,000	SAVR dominated TAVI SAVR vs MM $42,637 per QALY gained TAVI vs MM US$44,384 per QALY gained	Their model was sensitive to variations in the probabilities of *peri*-operative, annual mortality after each intervention, and the probability of annual stroke following AVR.
Gada et al 2012[34]	USA/Perspective of health care funding body / Funding not reported	Single Markov model	TF	TAVI SAPIEN vs MM and SAVR	Lifetime	Base case TAVI: US$59, 503 SAVR: US$56, 339 PARTNER A scenario TAVI: US$85, 513 SAVR: US$82, 989 PARTNER COSTS TAVI: US$81, 446 SAVR: US$79, 526	USD		TAVI:1.78 PARTNER A:1.78 PARTNER:2.14 AVR 1.72 PARTNER A:1.72 PARTNER B 2.15		US$100,000	TAVI vs MM $39,964 per QALY gained SAVR vs MM $39,280 per QALY gained Base case TAVI vs SAVR US$52, 773 per QALY gained PARTNER scenario US$252,400 per QALY gained PARTNER COST US$32, 000 per QALY gained	Model was sensitive t0 perioperative and annual mortality after AVR and after TAVI, and the probability of annual stroke after TAVI, are important determinants of the favored strategy
Neyt et al.23 2012[26]	Belgium/ Belgian Health Care Payer/ No funding was received	Single Markov model	TF/TA	TAVI SAPIEN vs SAVR SAPIEN	One year time horizon	Incremental costs:€20,397	EUR		Incremetnak gain: 0.03		€35, 000	Exceeding 750,000 per QALY gained	
Reynolds et al.2012[40]	USA/U.S. modified societal health care system perspective/ No funding reported	Trial-based	TF/TA	TAVI SAPIEN vs SAVR	One year time horizon	Incremental costs:$2,070 TAVI:$100,504 SAVR:$98,434 Transapical Incremental costs $11,896 TAVI $90,919 SAVR $79,024 TRANSFEMORAL Incremental costs:-$1250 Transfemoral TAVI $96,743 SAVR $97,992	USD	TAVI (TF-TA): 0.858 SAVR: 0.817 TAVI (TF): 0.878 SAVR: 0.813 TAVI (TA): 0.811 SAVR: 0.826	TAVI (TF-TA): 0.633 SAVR: 0.606 TAVI (TF): 0.659 SAVR: 0.591 TAVI (TA): 0.570 SAVR: 0.641		US$50000	TAVI (TF- TA) vs SAVR US$76,877 per QALY gained SAVR dominated Transapical TAVI Transfemoral TAVI dominated SAVR	

Abbreviations: TF, transfemoral; TA, transapical;SAVR, Surgical aortic valve replacement; MM, Medical Management

### 6.3 Intermediate risk-patients

Baron et al [46] proceeded to assess the financial profile of TAVI from a USA payer perspective in patients with intermediate risk, based on the PARTNER 2 trial [49]. Their analysis assessed both SAPIEN XT valve (XT-TAVR) and SAPIEN 3 valve vs SAVR. Efficacy data for the former were derived from the PARTNER 2A trial, while for the latter the PARTNER S3i registry was used. PARTNER S3i included an additional 1078 patients treated[50]. They employed a lifetime scenario, during total hospitalization costs were estimated to be marginally higher for XT-TAVR (P = 0.014) while they were $4,155 lower with S3-TAVR (P < 0.001) compared to TAVR. The follow-up costs were lower for both TAVI compared to SAVR (XT-TAVR (Δ=−$9,304; P < 0.001) and S3-TAVR (Δ=−$11,377; P < 0.001). regarding the overall TAVR was expected to contain total costs by $8,000 to $10,000, while an increase on quality-adjusted survival by 0.15 to 0.27 years was noted, which concluded that both valves dominated TAVR. Model was sensitive to TAVR-related follow-up costs.

Zhou et al evaluated the economic profile of SAPIEN 3 valve vs SAVR in intermediate risks population [48]. They created a Markov model, which was uploaded with data from PARTNER S3i study, with a 1—-year horizon. Costs were estimated from an Australian payer perspective. The utilities for the ‘Alive and well’ state were estimated from PARTNER S3i study. The ‘Alive with previous stroke’ utility was extrapolated from an Australian study on stroke survivors [32]. Other disutilities were calculated using a one-time factor. Authors reported that TAVI was interrelated to an incremental cost procedural costs $9,629 more than SAVR, driven primarily by the cost of the valve. TAVI delivered 0.33 more LYG and 0.31 QALY correspondingly. These render TAVI a dominant therapeutic option.

Goodall et al [49] performed an analysis from a French payer perspective through a MARKOV model, factoring the data of PARTNER II[50]. The model had a 15-year time horizon and it compared the cost-effectiveness of SAPIEN 3 vs SAVR. The use of TAVI dominated SAVR, resulting in incremental gains both in LYG and QALY (0.42 and 0.41 respectively) while their associated costs were lower (€439) TAVI dominated SAVR. The efficacy and health utilities used in the model were derived from the EQ-5D values reported in the PARTNER II study[51]. Model was sensitive to the admission costs for both SAVR and TAVI.

Kodera assessed the transfemoral implantation of TAVI with Sapien XT valve implantation vs SAVR in operable patients with intermediate surgical risk. Data were mined from the PARTNER 2 cohort. Model had a 10-year time horizon and the utilities were derived from PARTNER trials. Cost was set from the Japanese health payer perspective. In 10 years, the TAVI related costs outperformed the corresponding SAVR ones (¥8,039,694 vs ¥6,316,178 yen, respectively. TAVI delivered 0.22 QALY than SAVR (4.81 vs 4.59 QALYs). The ICUR of TAVI compared with SAVR was ¥7,523,821 yen per QALY gained, which exceeds the endorsed Japanese WTP. Model was sensitive to long-term mortality of TAVI[9].

Tam et al developed a fully probabilistic Markov model from the Canadian third-party payer’s perspective over a lifetime time horizon, in order the cost-effectiveness of the SAPIEN XT vs SAVR. Both therapeutic outcomes and costs were discounted at an annual rate of 1.5 %. The utilities were mined from PARTNER 1A trial while efficacy endpoints from the PARNTER 2 trial. TAVI was proved to yield more QALYs (an incremental gain of 0.23 QALYs was noted) albeit at an incremental cost of $10,547 CAD. The estimated ICUR/ QALY was $46,083 CAD, which constitutes TAVI a cost-effective option, while it was argued that results were sensitive to the cost of the TAVI prosthesis, complication rates and length of ICU stay [52].

Ribera evaluated the cost-effectiveness of transfemoral TAVI vs surgical replacement from a Spanish perspective, in intermediate -risk patients [53]. Their data set consisted from 207 patients: 58, 87 and 62 in the Edwards SAPIEN (ES) self-expandable TAVI, Medtronic-CoreValve (MC) self-expandable TAVI and SAVR groups respectively. The incremental cost of ES-TAVI vs SAVR was €8,800 and the corresponding incremental benefit was 0.036 QALY. This conferred an ICUR of €148,525/QALY. SAVR dominated the MC-TAVI, since MC was associated with higher costs €9,729 albeit at a bordeline lower QALY (−0.01). The results were sensitive to patients with high preoperative serum creatinine, cost of TAVI devices and hospitalization costs.

Pinar et al studied the economics of SAPIEN 3 TAVI from the Spanish Payer perspective. A Markov mathematical model with 9 exclusive states with monthly cycles was used based on the PARTNER 2 trial. Model had a 15-year time horizon and the utilities were extracted from the PARTNER 2 trial. Costs were set from the perspective of the Spanish health care system and they were discounted 3 % annually, along with benefits. TAVI yielded €50,950, €3,537 more than the commensurate ones of SAVR. The total LYG per TAVI patient was 6.08, as compared to 5.64 of SAVR patients, while the QALY gain was superior in TAVI patients (4.59 vs 4.15 QALY). The ICER perpetuates to €7,910/LYG and ICUR was €8,119/QALY [21].

**Fig. 4:**
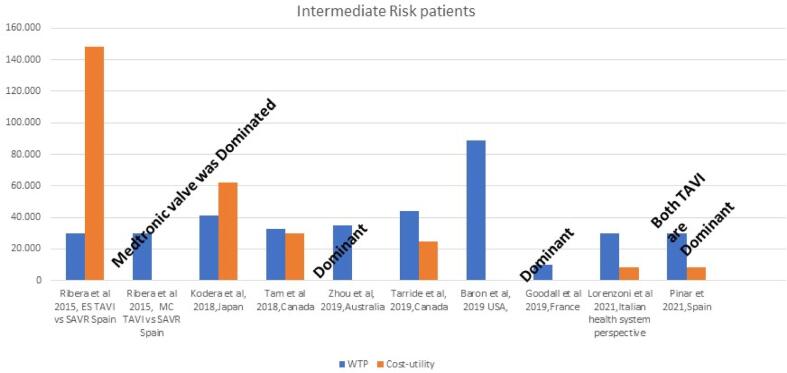
Cost-effectiveness results in intermediate-risk patients.

Lorenzoni estimated the cost-effectiveness of SAPIEN, from the perspective of the Italian National Health System (INHS) [24]. Regarding the intermediate risk patient cohort, a Markov model was created based on the survival data from the studies of Thourani et al and PARTNER 2 Leon et al. [10] for TAVI and SAVR, respectively. Costs were set from the perspective of the Italian Health care system and were discounted 3 % yearly, along with outcomes. TAVI delivered 4.21 QALY compared to 3.78 of TAVI, at an incremental cost of €3,593. TAVI was also associated with a gain 0.44 LYG. These led to an ICUR of €8,338 and an ICER of €8,035. The model was sensitive to mortality, to major incidence of stroke and repeated hospitalizations for AS. Tarride et al studied the economic profile of TAVI from a Canadian health care perspective [43]. They created a Markov Model with 9 mutually exclusive health states, comparing the SAPIEN 3 with SAVR, over a 15- year horizon. The model was loaded with the PARTNER 1A results and the utility studies from the PARTNER trial. The TAVI cohort reported more total costs (CAD$59,395 vS CAD$43,611) compared to the SAVR, while it delivered more QALYs (5.10 vS 4.62). This led to an ICUR of CAD$28,154/QALY. Model was sensitive to the time horizon of the study. (See Table 3 and Fig. 4).

**Table 3:** Economic evaluations in intermediate-risk patients.

**Author publication year)**	**Country/Perspective/ Funding**	**Agents**	**Time Horizon**	**Model**	**Treatment Incremental costs**	**Currency**	**LYG gained**	**QALY’s gained**	**Cost-effectiveness (CE) LYG**	**WTP**	**Cost-utility (CU) QALY**	**Other comments**
Lorenzoni et al 2021[24]	Italy/ Italian health system perspective/ Industry Funded	TAVI SAPIEN 3 vs SAVR	15-year time horizon	Markov Model with 8 states	Incremental costs: €3,593 TAVI: €36,623 SAVR: €33,030	EUR	Incremental gain:0.44 LYG TAVI: 6.08 LYG SAVR :5.64 LUG	Incremental gain: 0.43 QALY TAVI: 4.21 QALY SAVR: 3.78 QALY	€8,035 per LYG	€30,000	€8,338 per QALY gained	Model was sensitive to mortality, to major incidence of stroke and repeated hospitalizations for AS
Pinar et 2021[21]	Spain/ National Health Care System of Spain perspective/ Industry Funded	TAVI SAPIEN 3 SAVR	15-year time horizon	Markov mathematical model with 9 monthly exclusive states with	Incremental costs:€3,537 TAVI: €50,950 eur SAVR :€47,413	EUR	Incremental gain:0.44 LYG TAVI: 6.08 LYG SAVR: 5.64 LYG	Incremental gain:0.44 QALY TAVI: 4.59 QALY SAVR: 4.15 QALY	€7,910 per LYG	€30,000	€8,119 per QALY gained	
Zhou et al, 2019 [48]	Australia/Health Payer perspective/ Funded	TAVI vs SAVR	10-year time horizon	Markov Model	Incremental costs -$9,629 TAVI: $50,515 SAVR: $60,144	AUD	Incremental gain: 0.33 LYG TAVI:5.43 LYG SAVR:5.10 LYG	Incremental gain: 0.31 QALY TAVI:4.13 QALY SAVR:3,82 QALY	TAVI IS DOMINANT	$50,000	TAVI IS DOMINANT	Results were sensitive to SAVR length of stay and cost of TAVI
Baron et al USA,2019 [46]	USA/ US Healthcare system perspective/ Industry Funded	TAVI SAPIEN XT/ SAPIEN 3 vs SAVR	Lifetime	Markov Model	Incremental Costs: SAPIEN XT vs SAVR:-US$7949 SAPIEN 3 valve vs SAVR:-US$9692	USD	XT TAVR 7.80 (6.49 discounted) LYG SAVR 7.64(6.35 discounted) LYG S3-TAVR 7.95 (6.63 discounted) LYG SAVR 7.61 (6.34 discounted)LYG	XT TAVR:5.16 QALY SAVR: 5.01 QALY SAPIEN 3 5.29 QALY SAVR: 5.01		$100,000	BOTH TAVI DOMINATED SAVR	Model was sensitive to follow-up costs
Tarride et al,2019 [44]	Canada/Canadian third-party payer perspective/ Industry Funded	TAVI vs SAVR	15-year time horizon	Markov Model	Incremental costs: $15,784 TAVI:$59,395 SAVR:$43,611	USD	N/R	Incremental gain:0.48 TAVI: 5.10 SAVR:4.62		$50,000	$28,154 per QALY gained	Results were sensitive to the time-horizon of the study
Goodall et al 2019 [49]	France/French all payer perspective /Industry Funded	TAVI SAPIEN 3 vs SAVR	15-year time horizon		Incremental costs: -€439 for TAVI TAVI:€34,157 SAVR:€34,596	EUR	Incremental Gain: 0.42 LYG TAVI:5.87 SAVR:5.44	Incremental Gain:O.41 QALY TAVI:4.06 SAVR:3.65	TAVI dominates SAVR		TAVI dominates SAVR	Results were sensitive to TAVI and SAVR admission costs
Tam et al 2018 [52]	Canada/Canadian third-party payer’s perspective/ Industry Funded	TAVI SAPIEN XT vs SAVR	Lifetime	Markov Model	Incremental costs: $10,547 TAVI:$46,904 SAVR: $36,356	CAD	N/R	Incremental gain: 0.23 QALY TAVI:5.63 SAVR:5.40		$50,000	$46,083 per QALY gained	Resutls were sensitive to the acquisition cost and ICU length of stay
Kodera et al, 2018 [9]	Japan/Japanese public Healthcare payer/ No funding was received	TF TAVI vs SAVR	10 -year time horizon	Markov Model with Monte Carlo Simulations	Incremental costs: ¥1,723,516 ¥8,039,694 ¥6,316178	JPY	N/R	Incremental gain:0.22 QALY TAVI: 4.81 QALY SAVR:4.59 QALY	N/R	¥5,000,000	¥7,523,821 per QALY gained	Model was sensitive to long-term mortality of TAVI
Ribera et al 2015 [53]	Spain/ Spanish health services/Funded from Fondo de Investigación Sanitaria, Instituto Carlos III, and the Spanish Ministry of Economy and Competitiveness	TAVI Medtronic Corevalve(MC) vs Edward SAPIEN (ES) c vs SAVR	One-year time horizon	Costs were reported as mean (SD) and median and were compared using t-tests. Survival was assessed through Kaplan- Meier survival curves	ES-TAVI: €32,087 MC-TAVI: €32,111 SAVR:€ 23,288	EUR	N/R	ES-TAVI: 0.680 QALY MC-TAVI: 0.633 QALY SAVR: 0.644 QALY	N/R	€30,000	ES-TAVI vs SAVR: €148, 535 perQALY gained SAVR DOMINATED MC-TAVI	The results were sensitive to patients with high preoperative serum creatinine, cost of TAVR devices and hospitalization costs

Abbreviations: TF, transfemoral; TA, transapical; SAVR, Surgical aortic valve replacement

### 6.4 Intermediate-low risk

Kuntjoro assessed the cost-effectiveness of TAVI from the perspective of Singapore’s health system [54]. Their model consisted of two part: a short and a long-term phase model. The short-term phase model was a decision-tree one and covered the initial 30 days after procedure. This was followed by the long-term phase, which was a Markov model. The efficacy data were drawn from the PARTNER 2A trial[47]. Authors used direct costs from the perspective of the Singapore health system. The utilities were set according to the Singapore population norm for EuroQol-5D (EQ-5D) using local preference weights. Authors concluded that TF-TAVI was more effective than SAVR (3.86 vs 3.67 QALY) and also costlier (S$75,386 vs S$69,140) over an 8 year period. The generated ICUR was S$33,833/QALY. The model was sensitive to operational costs for both modalities.

### 6.5 Low risk

As more experience was collected in high and intermediate risk patient, a shift to low- risk patient was pondered. However, literature was bereft of data. The PARTNER 3 and the Evolut low risk trial spanned this gap and provided a realistic framework for exploring TAVI in this patient segment [55], [56], [57].

Gilard et al developed a four-state Markov model based on the PARTNER 3 trial, from the perspective of the France Health care system, based on a lifetime horizon. TAVI dominated SAVR, which was primarily attributed to the increased SAVR costs. The model was sensitive to the starting age of patients and the transition probabilities for treated AF and disabling stroke, for both interventions [58].

Tam published an economic evaluation of TAVI in a low-risk population from the perspective of the Canadian health care System, through the use of a fully probabilistic Markov Model, based on a network *meta*-analysis of the PARTNER 3 and Evolut low Risk Trials. The balloon-expandable TAVI yielded CAD$37,330, the self-expandable CAD$39,660 and SAVR CAD$34, 583. Respectively, the gains in terms of QALY was 9.15 ± 3.23, 9.13 ± 3.23, and 9.05 ± 3.20, respectively. The ICURs for balloon-expandable TAVR and self-expandable TAVR against SAVR were CAD$27,196/ QALY and CAD$59,641/QALY, respectively. Balloon-expandable TAVI dominated the self-expandable TAVI. Notably, the model was engulfed in significant uncertainty [59], which was primarily attributed to the changes in complication rates in both the TAVI and SAVR arm.

Geisler assessed the economic profile of TAVI vs SAVR in patients at low surgical risk from a Danish payer perspective [61]. They created a Markov state transition model based on data from the NOTION trial. The base case scenario concluded that TAVY was related with an incremental cost of DKK65,000 compared to SAVR (276,142 vs 211,581). Patient on TAVI patients also gained 0.09 more QALY than the SAVR cohort (QALYs; 5.39 vs 5.30). This perpetuates to an ICUR of DKK696,264/QALY (approximately €72,100/QALY).Given that the WTP threshold in Denmark is DKK1.1 million/QALY, TAVI is considered as a cost-effective option in Denmark. The model was sensitive to mortality risk for SAVR beyond the 48-month trial observation period, periprocedural mortality during TAVI, procedure costs for TAVI, length of stay for SAVR and TAVI index hospitalization, and the effectiveness discount rate.

**Fig. 5:**
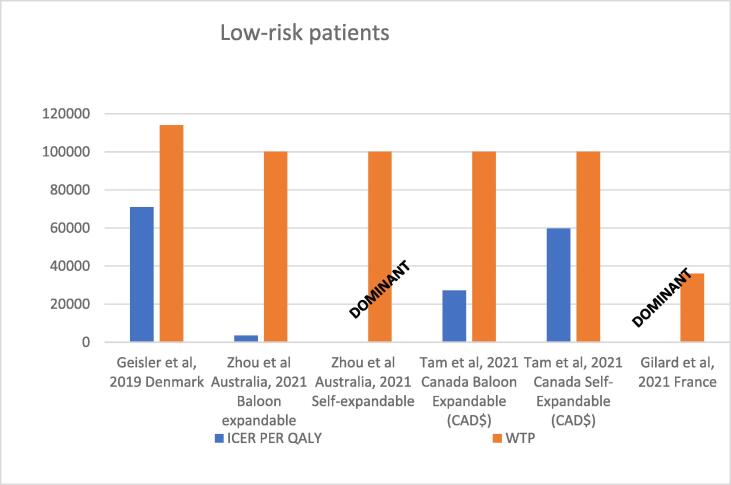
Cost-effectiveness results in low-risk patients.

Zhou conducted a cost-effectiveness analysis of TAVI vs SAVR from the perspective of the Australian Healthcare system, through a Markov model which compared TAVI to SAVR over a lifetime horizon and was based on the PARTNER 3 and Evolut Low-Risk trial for self-expanding TAVI [61]. Costs were obtained from Australian sources. The long-term mortality was calculated based on the Australian life tables. The utility values were estimated following the corresponding reports of the PARTNER S 3i intermediate risk study[61]. Authors concluded that the balloon expandable TAVI yields slightly higher total lifetime costs compared to SAVR over a lifetime scenario. (AU$61,259 vs AU$60,557). The balloon expandable was also more effective, both in terms of LYG and QALY gained as well (9.57 LYG vs 9.40, 7.40 QALY vs 7.20). The self-expandable valve dominated SAVR over a lifetime scenario. The model was sensitive to the ICU costs[62]. (See Table 4 and Fig. 5).

**Table 4:** Economic evaluations in low-risk patients.

**Author publication year)**	**Country/perspective/Funding**	**Access**	**Agents**	**Time Horizon**	**Model**	**Treatment Incremental costs**	**LYG gained**	**Currency**	**QALY’s gained**	**Cost-effectiveness (CE) LYG**	**WTP**	**Cost-utility (CU) QALY**	**Other comments**
Tam et al, 2021 [59]	Canada/Canadian third-party payer’s perspective/ Industry funded	TF	TAVI SAPIEN 3, TAVI Evolut R/ Evolut Pro or SAVR,	Life-time Horizon	Fully probabilistic Markov Model	SAPIEN 3; $37,330 EVOLUT; $39,660 SAVR; $34, 583	N/R	CAD	SAPIEN 3: 9.15 QALY EVOLUT: 9.13 QALY SAVR 9.05: QALY	N/R	$50 000 and $100 000	SAPIEN vs SAVR $27 196 per QALY gained EVOLUT vs SAVR: $59,641 per QALY gained	
Zhou et al, 2021[62]	Australia/Australian health care system perspective /Funded	TF	1)Balloon expandable vs SAVR 2)Self-expandable vs SAVR.	Life-time Horizon	Markov Model	Ballon expandable; $61,259 SAVR; $60,557 Self expandable TAVI; $64,585 SAVR; $65,093	Ballon expandable 9.57 LYG SAVR 9.40 LYG Self expandable TAVI 8.88 LYG SAVR 8.82 LYG	AUS	Ballon expandable: 7.40 QALY SAVR: 7.20 QALY Self expandable: 6.60 QALY SAVR :6.53 QALY	ICER of balloon-expandable vs SAVR $4,521 per LYG SELF EXPANDABLE DOMINATES SAVR	$100,000	ICUR of balloon-expandable vs SAVR: $3,521 per QALY gained SELF EXPANDABLE DOMINATES SAVR	Model was sensitive to ICU costs
Gilard et al, 2021 [58]	France/French national hospital claim perspective / Industry Funded	TF	SAPIEN 3 vs SAVR	Life-time Horizon	Markov Model	Incremental cost:€12,742 TAVI: €38 992 SAVR: €51,734	Incremental gain:0.8 LYG TAVI:14.07 LYG SAVR:13.18 LYG	EUR	Incremental gain: 0.89 QALY TAVI:8.44 QALY SAVR: 7.55 QALY	TAVI IS DOMINANT		TAVI IS DOMINANT	The model was sensitive to the starting age of patients and the transition probabilities toward treated AF and disabling stroke for both technologies
Geisler et al, 2019 [60]	Denmark/Danish societal perspective /Funded	TF	Core valve self expandable vs SAVR	Life-time Horizon	Markov state transition model, nested in a decision tree	Incremental cost: DKK 64,561 TAVI:DKK 276,142 SAVR:DKK 211,581	N/R	Denmark Koron	Incremental gain:0.09 QALY TAVI: 5.39 QALY SAVR: 5.30 QALY	N/R	DKK1.1 million/QALY	DKK696,264 per QALY gained	The model was sensitive to mortality risk for SAVR beyond the 48-month trial observation period, periprocedural mortality during TAVI, procedure costs for TAVI, length of stay for SAVR and TAVI index hospitalization, and the effectiveness discount rate.

Abbreviations: TF, transfemoral; TA, transapical; SAVR, Surgical aortic valve replacement

### 6.6 Sapiens vs Evolut

Veulemans et al proceeded to compare the economic profiles of Sapiens and Evolut. They retrospectively mined data from 204 patients in Germany. Nevertheless, this study fell short of meeting the inclusion criteria.

### 6.7 Quality

The majority of the studies demonstrated good methodological quality, as attested by the use of QHES tool, which bolsters their potential contribution in the decision-making process. QHES tool consists of 16 criteria and each one is assigned a binary weighted point value. The highest quality score for a study is 100 points. Although there is no explicit threshold to define characterize high quality studies, a study is considered to be of good quality if the score is > 75 points.

It also demonstrates good test–retest reliability of 0.95 (95 % CI 0.75–0.99) [63]. (See Table 5, Table 6, Table 7, Table 8).

**Table 5:** QHES for inoperable patients.

		Brecker et al, 2014	Doble et al 2013	Hancock et al 2013	INOUE et al 2020	Kodera et al 2018	Lorenzoni et al 2021	Murphy et al 2013	Pinar et al 2021	Watt et al 2011	Simons et al 2013	Neyt et al 2012	Reynolds et al 2012
1) Was the study objective presented in a clear, specific, and measurable manner?	7	7	7	7	7	7	7	7	7	7	7	7	7
2) Were the perspective of the analysis (societal, third-party payer, etc.) and reason for its selection stated?	4	4	4	4	4	4	4	4	4	4	4	4	4
3) Were variable estimates used in the analysis from the best available source (i.e.Randomized Control Trial –Best, Expert Opinion –Worst)?	8	8	8	8	8	8	8	8	8	8	8	8	8
4) If estimates came from a subgroup analysis, were the groups prespecified at the beginning of the study?	1	1	1	1	1	1	1	1	1	1	1	1	1
5) Was uncertainty handled by: 1) statistical analysis to address random events; 2) sensitivity analysis to cover a range of assumptions?	9	9	9	9	9	9	9	–	9	9		9	9
6) Was incremental analysis performed between alternatives for resources and costs?	6	6	6	6	6	6	6	6	6	6	6	6	6
7) Was the methodology for data abstraction (including the value of health states and other benefits) stated?	5	5	5	5	5	5	5	5	5	5	5	5	5
8) Did the analytic horizon allow time for all relevant and important outcomes? Were benefits and cost that went beyond one year discounted and a justification given for the discount rate?	7	–	7	7	7	7	7	–	7	7	7	7	7
9) Was the measurement of costs appropriate and the methodology for the estimation of quantities and unit costs clearly described?	8	8	8	8	8	8	8	8	8	8	8	8	8
10) Were the primary outcome measure(s) for the economic evaluation clearly stated and were the major short-term, long-term, and negative outcomes included?	6	6	6	6	6	6	6	6	6	6	6	6	6
11) Were the health outcomes measures/scales valid and reliable? If previously tested, valid and reliable measures were not available, was justification given for the measures/scale and reliable measures were not available, was justification given for the measures/scale used?	7	7	7	7	7	7	7	7	7	7	7	7	7
12) Were the economic model (including structure), study methods and analysis, and the components of the numerator and denominator displayed in a clear transparent manner?	8	8	8	8	8	8	8	8	8	8	8	8	8
13 Were the choice of economic model, main assumptions and limitations of the study stated and justified?	7	7	7	7	7	7	7	7	7	7	7	7	7
14) Did the author(s) explicitly discuss direction and magnitude of potential biases?	6	6	6	6	6	6	6	6	6	6	6	6	6
15) Were the conclusion/recommendations of the study justified and based on the study results?	8	8	8	8	8	8	8	8	8	8		8	8
16) Was there a statement disclosing the source of funding for the study?	3	3	3	3	3	3	3	3	3	3	3	3	3
**TOTAL SCORE**	**100**	**93**	**100**	**100**	**100**	**100**	**100**	**84**	**100**	**100**	**83**	**100**	**100**

**Table 6:** QHES FOR High risk patients.

		Doble et al	Fairbairn et al	Freeman et al	Gada et al 2012 Transapical	Gada et al 2012 TF	Geisler et al	Inoue et al	Lorenzoni et al	Orlando et al, 2013	Neyt et al 2012	Pinar et al 2021	Povero et al 2018	Reynold et al 2016	Tarride et al 2019	Reynolds et al, 2012
1) Was the study objective presented in a clear, specific, and measurable manner?	7	7	7	7	7	7	7	7	7	7	7	7	7	7	7	7
2) Were the perspective of the analysis (societal, third-party payer, etc.) and reason for its selection stated?	4	4	4	4	4	4	4	4	4	4	4	4	4	4	4	4
3) Were variable estimates used in the analysis from the best available source (i.e.Randomized Control Trial –Best, Expert Opinion –Worst)?	8	8	8	–	8	8	8	8	8	8	8	8	8	8	8	8
4) If estimates came from a subgroup analysis, were the groups prespecified at the beginning of the study?	1	1	1	1	1	1	1	1	1	1	1	1	1	1	1	1
5) Was uncertainty handled by: 1) statistical analysis to address random events; 2) sensitivity analysis to cover a range of assumptions?	9	9	9	–	9	9	9	9	9	9	9	9	9	9	9	9
6) Was incremental analysis performed between alternatives for resources and costs?	6	6	6	6	6	6	6	6	6	6	6	6	6	6	6	6
7) Was the methodology for data abstraction (including the value of health states and other benefits) stated?	5	5	5	5	–	–	5	5	5		5	5	5	5	5	5
8) Did the analytic horizon allow time for all relevant and important outcomes? Were benefits and cost that went beyond one year discounted and a justification given for the discount rate?	7	7	7	7	–	–	7	7	7	7	7	7	7	7	7	–
9) Was the measurement of costs appropriate and the methodology for the estimation of quantities and unit costs clearly described?	8	8	8	8	8	8	8	8	8	8	8	8	8	8	8	8
10) Were the primary outcome measure(s) for the economic evaluation clearly stated and were the major short-term, long-term, and negative outcomes included?	6	6	6	6	6	6	6	6	6	6	6	6	6	6	6	6
11) Were the health outcomes measures/scales valid and reliable? If previously tested, valid and reliable measures were not available, was justification given for the measures/scale and reliable measures were not available, was justification given for the measures/scale used?	7	7	7	7	7	7	7	7	7	7	7	7	7	7	7	7
12) Were the economic model (including structure), study methods and analysis, and the components of the numerator and denominator displayed in a clear transparent manner?	8	8	8	–	8	8	8	8	8	8	8	8	8	8	8	8
13 Were the choice of economic model, main assumptions and limitations of the study stated and justified?	7	7	7	7	7	7	7	7	7	7	7	7	7	7	7	7
14) Did the author(s) explicitly discuss direction and magnitude of potential biases?	6	6	6	6	6	6	6	6	6	6	6	6	6	6	6	6
15) Were the conclusion/recommendations of the study justified and based on the study results?	8	8	8	8	8	8	8	8	8	8	8	8	8	8	8	8
16) Was there a statement disclosing the source of funding for the study?	3	3	3	–	–	–	3	3	3	3	3	3	3	3	3	–
**TOTAL SCORE**	**100**	**100**	**100**	**72**	**85**	**85**	**100**	**100**	**100**	**95**	**100**	**100**	**100**	**100**	**100**	**90**

**Table 7:** QHES for intermediate risk patients.

	**Points**	Baron et al	Goodall et al	Kodera et al	Lorenzonie et al	Pinar et al	Ribera et al	Tam et al	Tarride et al	Zhou et al
1) Was the study objective presented in a clear, specific, and measurable manner?	7	7	7	7	7	7	7	7	7	7
2) Were the perspective of the analysis (societal, third-party payer, etc.) and reason for its selection stated?	4	4	4	4	4	4	4	4	4	4
3) Were variable estimates used in the analysis from the best available source (i.e.Randomized Control Trial –Best, Expert Opinion –Worst)?	8	8	8	8	8	8	–	8	8	8
4) If estimates came from a subgroup analysis, were the groups prespecified at the beginning of the study?	1	1	1	1	1	1	1	1	1	1
5) Was uncertainty handled by: 1) statistical analysis to address random events; 2) sensitivity analysis to cover a range of assumptions?	9	9	9	9	9	9	–	9	9	9
6) Was incremental analysis performed between alternatives for resources and costs?	6	6	6	6	6	6	6	6	6	6
7) Was the methodology for data abstraction (including the value of health states and other benefits) stated?	5	5	5	5	5	5	5	5	5	5
8) Did the analytic horizon allow time for all relevant and important outcomes? Were benefits and cost that went beyond one year discounted and a justification given for the discount rate?	7	7	7	7	7	7	7	7	7	7
9) Was the measurement of costs appropriate and the methodology for the estimation of quantities and unit costs clearly described?	8	8	8	8	8	8	8	8	8	8
10) Were the primary outcome measure(s) for the economic evaluation clearly stated and were the major short-term, long-term, and negative outcomes included?	6	6	6	6	6	6	6	6	6	6
11) Were the health outcomes measures/scales valid and reliable? If previously tested, valid and reliable measures were not available, was justification given for the measures/scale and reliable measures were not available, was justification given for the measures/scale used?	7	7	7	7	7	7	7	7	7	7
12) Were the economic model (including structure), study methods and analysis, and the components of the numerator and denominator displayed in a clear transparent manner?	8	–	8	8	8	8	8	8	8	8
13 Were the choice of economic model, main assumptions and limitations of the study stated and justified?	7	7	7	7	7	7	7	7	7	7
14) Did the author(s) explicitly discuss direction and magnitude of potential biases?	6	6	6	6	6	6	–	6	6	6
15) Were the conclusion/recommendations of the study justified and based on the study results?	8	8	8	8	8	8	8	8	8	8
16) Was there a statement disclosing the source of funding for the study?	3	3	3	3	3	3	3	3	3	3
**TOTAL SCORE**	**100**	**92**	**100**	**100**	**100**	**100**	**77**	**100**	**100**	**100**

**Table 8:** QHES for low-risk patients.

		Tam et al 2021	Zhou et al 2021	Geisler et al 2019	Gilard et al 2021
1) Was the study objective presented in a clear, specific, and measurable manner?	7	7	7	7	7
2) Were the perspective of the analysis (societal, third-party payer, etc.) and reason for its selection stated?	4	4	4	4	4
3) Were variable estimates used in the analysis from the best available source (i.e.Randomized Control Trial –Best, Expert Opinion –Worst)?	8	8	8	8	8
4) If estimates came from a subgroup analysis, were the groups prespecified at the beginning of the study?	1	1	1	1	1
5) Was uncertainty handled by: 1) statistical analysis to address random events; 2) sensitivity analysis to cover a range of assumptions?	9	9	9	9	9
6) Was incremental analysis performed between alternatives for resources and costs?	6	6	6	6	6
7) Was the methodology for data abstraction (including the value of health states and other benefits) stated?	5	5	5	5	5
8) Did the analytic horizon allow time for all relevant and important outcomes? Were benefits and cost that went beyond one year discounted and a justification given for the discount rate?	7	7	7	7	7
9) Was the measurement of costs appropriate and the methodology for the estimation of quantities and unit costs clearly described?	8	8	8	8	8
10) Were the primary outcome measure(s) for the economic evaluation clearly stated and were the major short-term, long-term, and negative outcomes included?	6	6	6	6	6
11) Were the health outcomes measures/scales valid and reliable? If previously tested, valid and reliable measures were not available, was justification given for the measures/scale and reliable measures were not available, was justification given for the measures/scale used?	7	7	7	7	7
12) Were the economic model (including structure), study methods and analysis, and the components of the numerator and denominator displayed in a clear transparent manner?	8	8	8	8	8
13 Were the choice of economic model, main assumptions and limitations of the study stated and justified?	7	7	7	–	7
14) Did the author(s) explicitly discuss direction and magnitude of potential biases?	6	6	6	6	6
15) Were the conclusion/recommendations of the study justified and based on the study results?	8	8	8	8	8
16) Was there a statement disclosing the source of funding for the study?	3	3	3	3	3
**TOTAL SCORE**	**100**	**100**	**100**	**93**	**100**

## 7 Discussion

This systematic review aims to update the existing body of evidence pertaining to the economics of TAVI across all patient cohorts.

As current evolutions in the medical sector increase life-expectancy, a collateral effect is the increasing prevalence of the elderly patients presenting with comorbidities. Therefore, new and safer interventions are explored, since these patients may not be amenable to the established surgical interventions. To this end, it is imperative to elucidate the economic profile of TAVI in this context, given that for certain patients no feasible alternative option apply. Indeed, the breadth of the alternative treatment modalities is rather scarce, especially in the inoperable cohort, which is demarcated by a staggering death rate, up to 50 %. Even for patients that are eligible for surgery cohorts, the risks of a cardiopulmonary bypass surgery must be also put into perspective as well.

Therefore, TAVI has attained a principal role in the aortic stenosis treatment context. The PARTNER (Cohorts A and B) and CoreValve trials (in high and extreme surgical risk patients) ascertain a significant improvement in the management of ‘inoperable’ patients, and an alternative in the high surgical risk patient. Nevertheless, the cost of the technology emerges as an important barrier, an aspect that dictates accurate and updated economic evaluations.

### 7.1 Inoperable patients

Our results annotate that in the cohort of inoperable patients, TAVI constitutes a proper therapeutic approach and the presented incremental costs streamline with the incremental health gains. We also observed that recent trials demonstrate a better cost-effectiveness profile, which can be attributed to improvement in operational procedures. Nevertheless, the co-morbidity of these patients must also be considered since the death rate is attributed to the general underlying health conditions, and not exclusively on the AS. Therefore, a thorough selection of patients must precede the decision of performing a TAVI. This entails the assessment of futility, which is a controversial topic. In this context, a thorough assessment of severe frailty, especially scrutinizing on issues such as prior stroke, moderate or severe dementia, or severe chronic obstructive pulmonary disease. The correct identification of these patients is crucial for the correct utilization of economic resources.

### 7.2 High-risk patients

In the next important patient cohort, high-risk patients, the use of TAVI represents a debatable choice. Results are inconsistent and inconclusive. The results oscillate and indicatively, in many studies TAVI was dominated or found to be more effective but too expensive relative to SVAR. The sensitivity analyses of the included studies underlined which factors are critical in safeguarding the cost-effectiveness of TAVI. Factors such as aortic regurgitation, paravalvular leakage and stroke have been ascertained. Therefore, the technological advances should be directed towards the design and optimization of the devices in order to demand smaller access, which in return will compound the aforementioned side effects. Both procedures are comparable in terms of efficacy and safety but the evidence is inconclusive from an economic point of view.

### 7.3 Intermediate- risk patients

The same inconclusive conclusion was reverberated in the intermediate risk patient cohort. Data deduce that TAVI is interlaced with a positive cost-effectiveness outcome, with results ranging from dominant, better and cheaper, to dominated, more expensive and inferior. Nevertheless, the devil is in the details and as described earlier, an array of variables influenced the outcome and may hinder an unconditional extrapolation without adaptation. Models were sensitive to long-term mortality of TAVI, high preoperative serum creatinine, SAVR length of stay cost of TAVI devices and hospitalization costs, probability of annual stroke following SAVR and time horizon of the study. Results are rather inconclusive and a personalized approach, formed by the characteristics of each patient should be the decisive factor. The time horizon was a key factor. Although a lifetime horizon was frequently used, the life expectancy of these patients may allow the use of a 10 or 15 year horizon. As attested by Neyt, shorter horizons escalated to burgeoning ICER.

### 7.4 Low-risk patients

As we transcend in the risk scale in the low-risk patients, the low number of studies, coupled with the marginal differences in terms of effectiveness, call out for a more in-depth assessment. In principal, this is echoed by Geisler et al who underlined -in their low-risk evaluation study-that results can be extrapolated only in countries with similar direct medical costs, due to the sensitivity of the model on the long-term mortality.

These findings align with previous systematic reviews, both regarding the inconclusiveness of data and also the proper and thorough selection of patients, which will be benefited from this technology [64], [65].

## 8 Access route and balloon vs self-expandable

We should underline that the stratification of TAVI by access route (TF or TA) demonstrated a substantial impact on its cost-effectiveness profile. Specifically, compared with SAVR, the TF-TAVI approach was cost-effective, with lower one-year costs and greater QALY gains, whereas the TA-TAVI approach was associated with higher costs and no clear improvement in quality of life, and thus was dominated by SAVR. Both TF and TA TAVI generated higher procedural costs compared with SAVR, which were attributed to the higher valve acquisition costs; however, TF-TAVI resulted in greater reductions in the length of stay compared with SAVR than did TA-TAVI, which offset the higher procedural costs and thus resulted in an improved ICUR. This was in particular underscored by Gada et al. Regarding the debate balloon vs self-expandable, only scarce comparative data exists, which hinder any potential comparison.

The notion of centers of excellence must be also put forward. It is anticipated that a certain level of expertise will help mitigate complication related to the procedure. Several countries have set a minimum number of TAVI implantations (50 per year) in order to meet the quality standards. Moreover, several key metrics should be monitored such as 30-day risk-adjusted all-cause mortality; 30-day all-cause neurologic events, including transient ischemic attack; 30-day major vascular complication; 30-day major bleeding; and 30-day moderate or severe aortic regurgitation and one year mortality and functional improvement. [66].

Therefore, the following conclusions have been reached:

1) The right patient must be identified. Eligible patients should clearly benefit from a less invasive intervention.
2) To this direction, risk scores should be improved in order to adequately capture and broadcast risk and the device cost as well.

The gaps in the literature need to be filled, especially with regards to mechanical and biological valves. Therefore, a proper selection of alternative modalities, may construe a more realistic and real-life applicable scenario. From a methodological perspective, almost all studies incorporated a Markov model and decision trees. The addition of patient-simulation models can integrate recurrent events and also remember previous health states of the patients, without rendering the program inflexible. In this direction, more subgroup analyses would be beneficial. In our *meta*-analysis, only a couple of studies utilized real-world data. We anticipate that economics evaluations, based on real-world data my offer another perspective on the economics of TAVI. However, real world data may be confounded by selection bias especially for the inoperable patient cohort.

## 9 Methodological perspective

The included studies employed a magnitude of economic models. In the inoperable patient cohort, the majority of the models employed Markov Models and decision analytic models. The lifetime horizon was ranging from 1-year to a lifetime horizon, with the majority utilizing longer horizon. As we transcend in the risk scale, studies tended to have longer durations. The majority employed a healthcare perspective.

The procurement cost of the technology must also be in the spotlight. Currently, a bundle of TAVI products are commercially available and in this perspective, market power is shifted at the payer’s site. Consequently, payers should negotiate the prices, given that the potential patient pool substantially expands. In the context of price negotiations, health agencies must capitalize on the accessibility of physicians to this modality and the indications for lower risk patient cohorts. In any case, these emerging features of this market must be incorporated in the reimbursement framework and broadcasted by the final decision as well.

## 10 Limitations of the study

Some of the included studies, did not fully present their economic models and the underlying assumptions. Moreover, the use of different valves, with regards to upgraded model, constitutes a confounding variable. The steep learning curve, may have been achieved earlier in certain countries, through the establishment the context of centers of excellence, and therefore, the real life results, may diverge from the reported ones. Also, we have observed that time horizon is a key factor pertinent to the outcome. In the inoperable risk cohort, the definition of MM is not the same across all included studies. Finally, the sensitivity analysis highlights a bullet of factors that may influence the outcome, and no clear conclusions can be drawn.

The dissonance engulfing the economic profile of TAVI does not seem to abate, as our data suggest.

## Declaration of Competing Interest

Dr P. Petrou is an employee of Cyprus Health Insurance Organisation (HIO). The views and opinions expressed in this publication are those of the author. They do not purport to reflect the opinions or views of the HIO. No funding was received for this study.

## References

[1] Ben-Dor I., Pichard A., Gonzalez M., Weissman G., Li Y., Goldstein S. (2010). Correlates and causes of death in patients with severe symptomatic aortic stenosis who are not eligible to participate in a clinical trial of transcatheter aortic valve implantation. Circulation.

[2] Cribier A., Eltchaninoff H., Bash A., Borenstein N., Tron C., Bauer F., Derumeaux G., Anselme F., Laborde F., Leon M.B. (2002 Dec 10). Percutaneous transcatheter implantation of an aortic valve prosthesis for calcific aortic stenosis: first human case description. Circulation.

[3] Bevan G.H., Zidar D.A., Josephson R.A., Al-Kindi S.G. (2019). Mortality due to aortic stenosis in the United States, 2008–2017. JAMA.

[4] Forrest J.K. (2012). Transcatheter aortic valve replacement: design, clinical application, and future challenges. Yale J. Biol. Med..

[5] Alec Vahanian, Friedhelm Beyersdorf, Fabien Praz, Milan Milojevic, Stephan Baldus, Johann Bauersachs, et al , ESC/EACTS Scientific Document Group, 2021 ESC/EACTS Guidelines for the management of valvular heart disease: Developed by the Task Force for the management of valvular heart disease of the European Society of Cardiology (ESC) and the European Association for Cardio-Thoracic Surgery (EACTS), European Heart Journal, 2021;, ehab395, https://doi.org/10.1093/eurheartj/ehab395.

[6] Page M.J., McKenzie J.E., Bossuyt P.M., Boutron I., Hoffmann T.C., Mulrow C.D. (2021). The PRISMA 2020 statement: an updated guideline for reporting systematic reviews. BMJ.

[7] Chiou C.F., Hay J.W., Wallace J.F. (2003). Development and validation of a grading system for the quality of cost-effectiveness studies. Med. Care.

[8] Ofman J.J., Sullivan S.D., Neumann P.J., Chiou C.F., Henning J.M., Wade S.W., Hay J.W. (Jan-Feb 2003). Examining the value and quality of health economic analyses: implications of utilizing the QHES. J. Manag. Care Pharm..

[9] Kodera S., Kiyosue A., Ando J., Komuro I. (2018 Mar). Cost effectiveness of transcatheter aortic valve implantation in patients with aortic stenosis in Japan. J. Cardiol..

[10] Leon M.B., Smith C.R., Mack M. (2010). Transcatheter aortic-valve implantation for aortic stenosis in patients who cannot undergo surgery. N. Engl. J. Med..

[11] Sachie Inoue, Koichi Nakao, Michiya Hanyu, Kentaro Hayashida, Hidetoshi Shibahara, Makoto Kobayashi, Miyoshi Asaoka Cost-Effectiveness of Transcatheter Aortic Valve Implantation Using a Balloon-Expandable Valve in Japan: Experience From the Japanese Pilot Health Technology Assessment Value in Health Regional Issues Vol 1 , P82-90, MAY 01, 2020. 10.1016/j.vhri.2019.07.013 31670112

[12] Makkar R.R., Fontana G.P., Jilaihawi H. (2012). Transcatheter aortic-valve replacement for inoperable severe aortic stenosis. N. Engl. J. Med..

[13] Reynolds M.R., Magnuson E.A., Lei Y. (2011). Health-related quality of life after transcatheter aortic valve replacement in inoperable patients with severe aortic stenosis. Circulation.

[14] Rebecca L. Hancock-Howard, Christopher M. Feindel, Josep Rodes-Cabau, John G. Webb, Ann K. Thompson & Kurt Banz (2013) Cost effectiveness of transcatheter aortic valve replacement compared to medical management in inoperable patients with severe aortic stenosis: Canadian analysis based on the PARTNER Trial Cohort B findings, Journal of Medical Economics, 16:4, 566-574, DOI: 0.3111/13696998.2013.77074. 10.3111/13696998.2013.770747 23356420

[15] Watt M. (2012). Cost-effectiveness of transcatheter aortic valve replacement in patients ineligible for conventional aortic valve replacement. Heart.

[16] Murphy A. (2013). Transcatheter aortic valve implantation for severe aortic stenosis: the cost-effectiveness case for inoperable patients in the United Kingdom. Int. J. Technol. Assess. Health Care.

[17] Maliwa M., van der Heijden G., Bots M. (2003). Quality of life and NYHA class 30 years after mechanical aortic valve replacement. Cardiovasc. Surg..

[18] Simons C.T. (2013). Transcatheter aortic valve replacement in nonsurgical candidates with severe, symptomatic aortic stenosis: a cost-effectiveness analysis. Circulation.

[19] Brecker S. (2014). Cost-utility of transcatheter aortic valve implantation for inoperable patients with severe aortic stenosis treated by medical management: a UK cost-utility analysis based on patient-level data from the ADVANCE study. Open Heart.

[20] Linke A., Wenaweser P., Gerckens U. (2014). Treatment of aortic stenosis with a self-expanding transcatheter valve: the International multi-centre ADVANCE study. Eur. Heart J..

[21] Pinar E, García de Lara J, Hurtado J, Robles M, Leithold G, Martí-Sánchez B, Cuervo J, Pascual DA, Estévez-Carrillo A, Crespo C. Cost-effectiveness analysis of the SAPIEN 3 transcatheter aortic valve implant in patients with symptomatic severe aortic stenosis. Rev Esp Cardiol (Engl Ed). 2022 Apr;75(4):325-333. English, Spanish. doi: 10.1016/j.rec.2021.02.013. Epub 2021 May 18. PMID: 34016548. 10.1016/j.rec.2021.02.013 34016548

[22] Doble B., Blackhouse G., Goeree R., Xie F. (July 2013). Cost-effectiveness of the Edwards SAPIEN transcatheter heart valve compared with standard management and surgical aortic valve replacement in patients with severe symptomatic aortic stenosis: a Canadian perspective. J. Thorac. Cardiovasc. Surg..

[23] Matthew R. Reynolds, Elizabeth A. Magnuson, Kaijun Wang, Yang Lei, Katherine Vilain, Joshua Walczak, et al Cost-Effectiveness of Transcatheter Aortic Valve Replacement Compared With Standard Care Among Inoperable Patients With Severe Aortic Stenosis Results From the Placement of Aortic Transcatheter Valves (PARTNER) Trial (Cohort B) Circulation 125(9), 1102–1109 (2012). 10.1161/CIRCULATIONAHA.111.054072 22308299

[24] Lorenzoni V., Barbieri G., Saia F., Meucci F., Martinelli G.L., Cerillo A.G., Berti S., Candolf P., Turchetti G. (2021). The cost effectiveness of transcatheter aortic valve implantation: exploring the Italian National Health System perspective and different patient risk groups. Eur. J. Health Econ..

[25] Herrmann H.C., Thourani V.H., Kodali S.K., Makkar R.R., Szeto W.Y., Anwaruddin S.C., Kereiakes D.J., Ramee S., Greason K.L., Kapadia S. (2016). One-year clinical outcomes with SAPIEN 3 transcatheter aortic valve replacement in high-risk and inoperable patients with severe aortic stenosis. Circulation.

[26] Neyt M., Van Brabandt H., Devriese S., Van De Sande S. (2012 May 4). A cost-utility analysis of transcatheter aortic valve implantation in Belgium: focusing on a well-defined and identifiable population. BMJ Open..

[27] Schymik G, Lefèvre T, Bartorelli AL, et al. European experience with the second-generation Edwards SAPIEN XT transcatheter heart valve in patients with severe aortic stenosis: 1-year outcomes from the SOURCE XT Registry.JACC Cardiovasc Interv. 2015; 8:657–669. 10.1016/j.jcin.2014.10.026 25946437

[28] Deeb G.M., Reardon M.J., Chetcuti S. (2016). 3-year outcomes in high-risk patients who underwent surgical or transcatheter aortic valve replacement. J. Am. Coll. Cardiol..

[29] Fairbairn T.A., Meads D.M., Hulme C., Mather A.N., Plein S., Blackman D.J., Greenwood J.P. (2013 Jul). The cost-effectiveness of transcatheter aortic valve implantation versus surgical aortic valve replacement in patients with severe aortic stenosis at high operative risk. Heart.

[30] Freeman P.M. (2016). Severe symptomatic aortic stenosis: medical therapy and transcatheter aortic valve implantation (TAVI)— a real-world retrospective cohort analysis of outcomes and costeffectiveness using national data. Open Heart.

[31] Gada H, Agarwal S, Thomas H. Marwick Perspective on the cost-effectiveness of transapical aortic valve implantation in high-risk patients: Outcomes of a decision-analytic model Ann Cardiothorac Surg 2012;1(2):145-155. 10.3978/j.issn.2225-319X.2012.06.12 PMC3741749 23977485

[32] Wenaweser P., Pilgrim T., Kadner A. (2011). Clinical outcomes of patients with severe aortic stenosis at increased surgical risk according to treatment modality. J. Am. Coll. Cardiol..

[33] Sullivan P.W., Ghushchyan V. (2006). Preference-based EQ-5D index scores for chronic conditions in the United States. Med. Decis. Making.

[34] Gada H. (2012). Markov model for selection of aortic valve replacement versus transcatheter aortic valve implantation (without replacement) in high-risk patients. Am. J. Cardiol..

[35] Povero M., Miceli A., Pradelli L., Ferrarini M., Pinciroli M., Glauber M. (2018 Nov). Cost-utility of surgical sutureless bioprostheses vs TAVI in aortic valve replacement for patients at intermediate and high surgical risk. Clinicoecon. Outcomes Res..

[36] Wendler O., Schymik G., Treede H. (2017). SOURCE 3 registry: design and 30-day results of the European postapproval registry of the latest generation of the SAPIEN 3 transcatheter heart valve. Circulation.

[37] Ussia G.P., Barbanti M., Petronio A.S. (2012). Transcatheter aortic valve implantation: 3-year outcomes of self-expanding CoreValve prosthesis. Eur. Heart J..

[38] Kodali S.K., Williams M.R., Smith C.R. (2012). Two-year outcomes after transcatheter or surgical aortic-valve replacement. N. Engl. J. Med. Overseas Ed..

[39] Mack, M.J., Leon, M.B., Smith, C.R., Miller, D.C., Moses, J.W., Tuzcu, et al : 5-year outcomes of transcatheter aortic valve replacement or surgical aortic valve replacement for high surgical risk patients with aortic stenosis (PARTNER 1): a randomised controlled trial. Lancet (2015). https:// doi. org/ 10. 1016/ S0140- 6736(15) 60308-7. 10.1016/S0140-6736(15)60308-7 25788234

[40] Reynolds M.R. (2012). Cost-effectiveness of transcatheter aortic valve replacement compared with surgical aortic valve replacement compared with surgical aortic valve Cost Effectiveness of TAVR 43 replacement in high-risk patients with severe aortic stenosis: results of the PARTNER (placement of aortic transcatheter valves) trial (Cohort A). J. Am. Coll. Cardiol..

[41] Reynolds MR, Lei Y, Wang K, Chinnakondepalli K, Vilain KA, Magnuson EA, Galper BZ, Meduri CU, Arnold SV, Baron SJ, Reardon MJ, Adams DH, Popma JJ, Cohen DJ; CoreValve US High Risk Pivotal Trial Investigators. Cost-Effectiveness of Transcatheter Aortic Valve Replacement With a Self-Expanding Prosthesis Versus Surgical Aortic Valve Replacement. J Am Coll Cardiol. 2016 Jan 5;67(1):29-38. doi: 10.1016/j.jacc.2015.10.046. PMID: 26764063; PMCID: PMC4959424. 10.1016/j.jacc.2015.10.046 PMC4959424 26764063

[42] Geisler B.P., Huygens S.A., Reardon M.J., Van Mieghem N., Kappetein A.P., Osnabrugge R.L.J., Pietzsch J.B. (2017). Cost- effectiveness and projected survival of self-expanding transcatheter versus surgical aortic valve replacement for high-risk patients in a European setting: a Dutch analysis based on the CoreValve high risk trial. Structural Heart.

[43] Adams D.H., Popma J.J., Reardon M.J. (2014). Transcatheter aortic valve replacement with a self-expanding prosthesis. N. Engl. J. Med..

[44] Jean-Eric Tarride, Trinh Luong, Gordon Goodall, Natasha Burke, Gordon Blackhouse Canadian cost-effectiveness analysis of SAPIEN 3 transcatheter aortic valve implantation compared with surgery, in intermediate and high-risk severe aortic stenosis patients ClinicoEconomics and Outcomes Research 2019:11 477–486. 10.2147/CEOR.S208107 PMC6677373 31551658

[45] R Orlando, M Pennant, S Rooney, S Khogali, S Bayliss, A Hassan, D Moore and P Barton Cost-effectiveness of transcatheter aortic valve implantation (TAVI) for aortic stenosis in patients who are high risk or contraindicated for surgery: a model-based economic evaluation HEALTH TECHNOLOGY ASSESSMENT Volume 17 Issue 33 August 2013. 10.3310/hta17330 PMC4781377 23948359

[46] Suzanne J. Baron, Kaijun Wang, John A. House, Elizabeth A. Magnuson, Matthew R. Reynolds, Raj Makkar, Cost-Effectiveness of Transcatheter Versus Surgical Aortic Valve Replacement in Patients With Severe Aortic Stenosis at Intermediate Risk Circulation. 2019;139:877–888. DOI: 10.1161/CIRCULATIONAHA.118.035236. 10.1161/CIRCULATIONAHA.118.035236 30586747

[47] Leon M.B., Smith C.R., Mack M.J., Makkar R.R., Svensson L.G., Kodali S.K. (2016). PARTNER 2 investigators. transcatheter or surgical aortic-valve replacement in intermediate-risk patients. N. Engl. J. Med..

[48] Zhou J., Liew D., Duffy S.J., Walton A., Htun N. (2019). Dion Stub Cost-effectiveness of transcatheter aortic valve implantation compared to surgical aortic valve replacement in the intermediate surgical risk population. Int. J. Cardiol..

[49] Goodall G., Lamotte M., Ramos M., Maunoury F., Pejchalova B., de Pouvourville G. (2019). Cost-effectiveness analysis of the SAPIEN 3 TAVI valve compared with surgery in intermediate-risk patients. J. Med. Econ..

[50] Baron S.J., Arnold S.V., Wang K. (2017). Health status benefits of transcatheter vs surgical aortic valve replacement in patients with severe aortic stenosis at intermediate surgical risk: results from the PARTNER 2 randomized clinical trial. JAMA Cardiol..

[51] Thourani V.H., Kodali S., Makkar R.R. (2016). Transcatheter aortic valve replacement versus surgical valve replacement in intermediate-risk patients: a propensity score analysis. Lancet.

[52] Derrick Y. Tam, Avery Hughes, Stephen E. Fremes, Saerom Youn,Rebecca L. Hancock-Howard, Peter C. Coyte, A cost-utility analysis of transcatheter versus surgical aortic valve replacement for the treatment of aortic stenosis in the population with intermediate surgical risk The Journal of Thoracic and Cardiovascular Surgery. May 2018. 10.1016/j.jtcvs.2017.11.112 29454487

[53] Aida Ribera John Slof, , Rut Andrea, Carlos Falces, Enrique Gutiérrez , Raquel del Valle-Fernández. Transfemoral transcatheter aortic valve replacement compared with surgical replacement in patients with severe aortic stenosis and comparable risk: Cost–utility and its determinants International Journal of Cardiology 182 (2015) 321–328. 10.1016/j.ijcard.2014.12.109 25585368

[54] Ivandito Kuntjoro, Edgar Tay, Jimmy Hon, James Yip, William Kong, Kian Keong Poh Cost-Effectiveness of Transcatheter Aortic Valve Implantation in Intermediate and Low Risk Severe Aortic Stenosis Patients in Singapore Ann Acad Med Singapore 2020;49:423–33. 33000105

[55] Popma J.J., Deeb G.M., Yakubov S.J., Mumtaz M., Gada H., O’Hair D. (2019). Transcatheter aortic-valve replacement with a self-expanding valve in low-risk patients. N. Engl. J. Med..

[56] Thyregod H.G., Steinbruchel D.A., Ihlemann N., Nissen H., Kjeldsen B.J., Petursson P. (2015). Transcatheter versus surgical aortic valve replacement in patients with severe aortic valve stenosis: 1-year results from the all-comers NOTION randomized clinical trial. J. Am. Coll. Cardiol..

[57] Mack M.J., Leon M.B., Thourani V.H., Makkar R., Kodali S.K., Russo M. (2019). Transcatheter aortic-valve replacement with a balloon-expandable valve in low-risk patients. N. Engl. J. Med..

[58] Gilard M., Eltchaninoff H., Iung B., Lefèvre T., Spaulding C., Dumonteil N. (2022). Cost-Effectiveness Analysis of SAPIEN 3 Transcatheter Aortic Valve Implantation Procedure Compared With Surgery in Patients With Severe Aortic Stenosis at Low Risk of Surgical Mortality in France Value Health.

[59] Tam D.Y., Azizi P.M., Fremes S.E., Chikwe J., Harindra M.G., Wijeysundera C. (2021 Oct 28). The cost-effectiveness of transcatheter aortic valve replacement in low surgical risk patients with severe aortic stenosis. Eur Heart J Qual Care Clin Outcomes..

[60] Geisler BP, Jørgensen TH, Thyregod HGH, Pietzsch JB, Søndergaard L Cost-Effectiveness of Transcatheter versus Surgical Aortic Valve Replacement in Patients at Lower Surgical Risk: Results from the NOTION Trial. EuroIntervention 2019; Jaa-632 2019, doi: 10.4244/EIJ-D-18-00847. 10.4244/EIJ-D-18-00847 31422922

[61] Baron S.J., Thourani V.H., Kodali S., Arnold S.V., Wang K., Magnuson E.A. (2018). Effect of SAPIEN 3 transcatheter valve implantation on health status in patients with severe aortic stenosis at intermediate surgical risk: results from the PARTNER S3i trial. JACC Cardiovasc. Interv..

[62] Jennifer Y. Zhou, MBBS, Danny Liew, Stephen J. Duffy, Antony Walton, Nay Htun, , Dion Stub Cost-Effectiveness of Transcatheter Versus Surgical Aortic Valve replacement in Low-Risk Patients With Severe Aortic Stenosis. Heart, Lung and Circulation (2021) 30, 547–554. 10.1016/j.hlc.2020.09.934 33189571

[63] Gerkens S., Crott R., Cleemput I., Thissen J.-P., Closon M.-C., Horsmans Y., Beguin C. (2008). Comparison of three instruments assessing the quality of economic evaluations: A practical exercise on economic evaluations of the surgical treatment of obesity. Int. J. Technol. Assess. Health Care.

[64] Fotini Gialama Panagiotis Prezerakos , Vasilis Apostolopoulos , and Nikolaos Maniadaki Systematic review of the cost-effectiveness of transcatheter interventions for valvularheart disease European Heart Journal - Quality of Care and Clinical Outcomes (2018) 0, 1–10. 10.1093/ehjqcco/qcx049 29325012

[65] Andrea Iannaccone Thomas H. Marwick Cost Effectiveness of Transcatheter Aortic Valve Replacement Compared with Medical Management or Surgery for Patients with Aortic Stenosis Appl Health Econ Health Policy (2015) 13:29–45. 10.1007/s40258-014-0141-6 25488391

[66] Bavaria JE, Tommaso CL, Brindis RG, Carroll JD, Deeb GM, Feldman TE, Gleason TG, Horlick EM, Kavinsky CJ, Kumbhani DJ, Miller DC, Seals AA, Shahian DM, Shemin RJ, Sundt TM 3rd, Thourani VH. 2018 AATS/ACC/SCAI/STS Expert Consensus Systems of Care Document: Operator and Institutional Recommendations and Requirements for Transcatheter Aortic Valve Replacement: A Joint Report of the American Association for Thoracic Surgery, American College of Cardiology, Society for Cardiovascular Angiography and Interventions, and Society of Thoracic Surgeons. J Am Coll Cardiol. 2019 Jan 29;73(3):340-374. doi: 10.1016/j.jacc.2018.07.002. Epub 2018 Jul 18. Erratum in: J Am Coll Cardiol. 2018 Aug 3;: PMID: 30031107. 10.1016/j.jacc.2018.07.002 30031107

